# Neuroprotective protein ADNP-dependent histone remodeling complex promotes T helper 2 immune cell differentiation

**DOI:** 10.1016/j.immuni.2023.05.010

**Published:** 2023-07-11

**Authors:** Ana C.F. Ferreira, Aydan C.H. Szeto, Paula A. Clark, Alastair Crisp, Patrycja Kozik, Helen E. Jolin, Andrew N.J. McKenzie

**Affiliations:** 1 MRC Laboratory of Molecular Biology, Cambridge CB2 0QH, UK

**Keywords:** immunity, ADNP, T helper 2 cells, Th2, type 2 cytokines, IL-13, asthma, histone remodeling, AP-1, GATA3

## Abstract

Type 2 immune responses are critical in tissue homeostasis, anti-helminth immunity, and allergy. T helper 2 (Th2) cells produce interleukin-4 (IL-4), IL-5, and IL-13 from the type 2 gene cluster under regulation by transcription factors (TFs) including GATA3. To better understand transcriptional regulation of Th2 cell differentiation, we performed CRISPR-Cas9 screens targeting 1,131 TFs. We discovered that activity-dependent neuroprotector homeobox protein (ADNP) was indispensable for immune reactions to allergen. Mechanistically, ADNP performed a previously unappreciated role in gene activation, forming a critical bridge in the transition from pioneer TFs to chromatin remodeling by recruiting the helicase CHD4 and ATPase BRG1. Although GATA3 and AP-1 bound the type 2 cytokine locus in the absence of ADNP, they were unable to initiate histone acetylation or DNA accessibility, resulting in highly impaired type 2 cytokine expression. Our results demonstrate an important role for ADNP in promoting immune cell specialization.

## Highlights

• CRISPR screen identifies ADNP-mediated regulation of type 2 cytokine expression
• ADNP is required to promote type 2 cytokine production
• ADNP recruits the CHD4-BRG1 complex to remodel chromatin at the Th2 cytokine locus
• ADNP-deficient mice display impaired antigen-specific Th2 cell responses

## Graphical abstract

**Figure:**
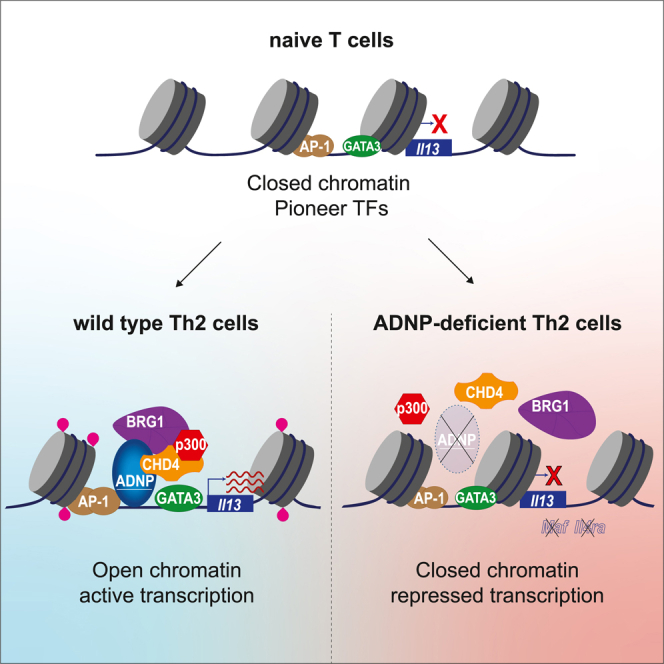

## Teaser

Th2 cells orchestrate type 2 immune responses by producing specific cytokines, but the mechanism underlying their regulation is not fully understood. Ferreira et al. discover that the neuroprotective protein ADNP is required for effective IL-13 production by Th2 cells and is indispensable for immune reactions to allergens.

## Introduction

To perform their roles, T lymphocytes and innate lymphoid cells (ILCs) must be able to differentiate in response to external stimuli to initiate specialized protective immune responses. For example, T cells and ILCs express interferon-γ promote so-called type 1 defenses against bacteria, viruses, and cancer, whereas fungi induce type 3 immunity.^1^ Type 2 immune responses are critical for tissue homeostasis, tissue repair, and protection of the host from infections such as parasitic helminths.^2^^,^^3^^,^^4^ However, inappropriate type 2 immunity can lead to asthma and allergy.^5^^,^^6^ The key cellular orchestrators of these responses are ILC2s and adaptive T helper 2 (Th2) cells, which produce the secreted type 2 effector cytokines interleukin-4 (IL-4), IL-5, and IL-13.^2^^,^^3^^,^^4^^,^^5^ These cytokines act as messengers to induce the widespread activation of immune effector cells such as eosinophils, mucus-producing goblet cells, B cells, T cells, and macrophages.^7^ Consequently, the precise regulation of type 2 lymphocytes and ILs is critical in health and disease.^6^

The type 2 cytokine gene locus comprises a region of ∼150 kb on mouse chromosome 11 (and human chromosome 5), which harbors the *Il4*, *Il5*, and *Il13* gene cluster, encoding IL-4, IL-5, and IL-13. During Th2 cell differentiation, this locus undergoes extensive chromatin remodeling and architectural reorganization.^8^^,^^9^^,^^10^ The pioneer transcription factor (TF) GATA3 is the “master regulator” of Th2 cell differentiation and type 2 IL expression^11^ and acts synergistically with other pioneer TFs such as the activator protein 1 (AP-1) factors JUNB and BATF,^12^^,^^13^ and TFs from the NF-κB and NFAT families.^14^ By binding gene regulatory regions, these factors promote the recruitment of histone-modifying enzymes (e.g., histone acetyl transferases [HATs]) and chromatin remodeling complexes, which reshape the local chromatin landscape.^15^^,^^16^^,^^17^ These processes increase chromatin accessibility for the recruitment of additional factors and promote transcription. Indeed, components of the ATPase-dependent BAF (BRG/BRM associated factor, mSWI/SNF) chromatin remodeling complex, for example, Brahma-related gene-1 (BRG1, encoded by *Smarca4*), play key roles in Th2 cell differentiation and type 2 cytokine production.^18^ Contributing to this activated Th2 cell phenotype, regulators of genome architecture and long-range chromosome interactions also play roles in Th2 cell differentiation.^8^^,^^19^ The DNA-binding protein CCCTC-binding factor (CTCF) which insulates topologically associated domains (TADs), and cooperates with the cohesin complex to form chromatin loops to bring together enhancers and/or silencers to regulate gene expression, is known to be required for type 2 cytokine expression.^20^ Further, the TF Ying Yang (YY1) was found to bind multiple regions in the type 2 cytokine locus before the recruitment of GATA3, to which it associates physically and induce DNA loop formation.^21^

Despite our knowledge that GATA3 is necessary for type 2 cytokine expression,^2^^,^^22^ in combination with TFs such as NF-κB, AP-1 and NFAT, and chromatin modifiers, we lack insight into how these transitions are coordinated and maintained in a Th2-cell-specific manner. To address this question, we performed a CRISPR-Cas9 screen in differentiating Th2 cells to identify factors that selectively regulated type 2 cytokine gene expression. This approach identified a mechanism by which activity-dependent neuroprotector homeobox protein (ADNP) has a critical role in promoting the Th2 cell phenotype.

## Results

### Identification of ADNP as a regulator of IL-13 expression by Th2 cells

**Figure 1:**
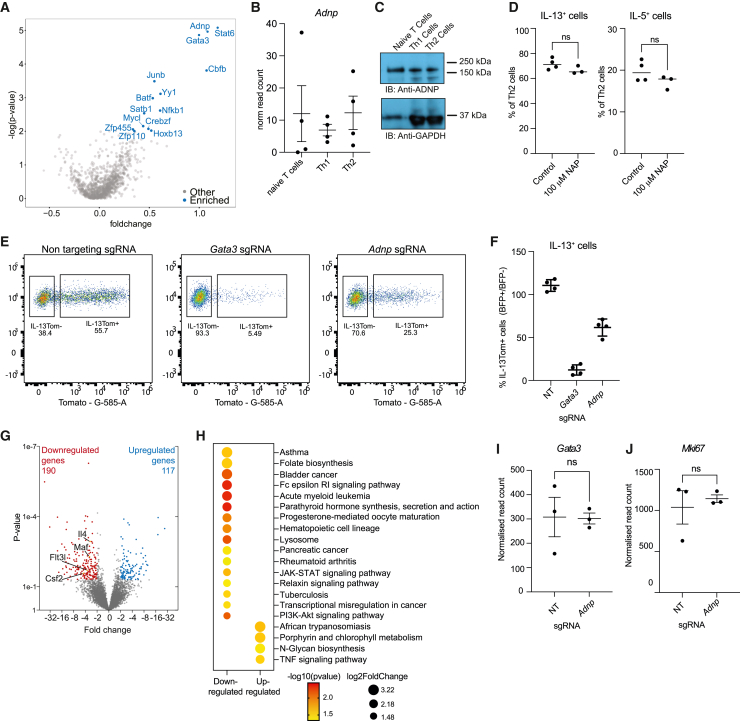
Identification of ADNP as a regulator of IL-13 expression by Th2 cells (A) Volcano plot showing positive regulators of Th2 cell differentiation (blue). Data are pooled from 2 independent screens. (B) *Adnp* gene expression (from RNA-seq analysis) in naive T cells, Th1, and Th2 cells. Mean ± SD. (C) Detection of ADNP (150 kDa) and GAPDH (36 kDa) proteins in T cell lysates. (D) Flow cytometric analysis of cytokine expression by Th2 cells cultured in the presence of vehicle (PBS) or NAP. Data are representative of 2 independent experiments; unpaired two-sided t test; not significant (ns). (E) Representative flow cytometry gating strategy for the analysis of IL-13Tom expression by Th2 cells transduced with sgRNAs. (F) Flow cytometric analysis of IL-13Tom expression by Th2 cells transduced with sgRNAs. NT, non-targeting. Mean ± SD. (G) Volcano plot showing RNA sequencing analysis of *Adnp* sgRNA-targeted versus non-targeted Th2 cells. (H) KEGG pathway analysis of genes in *Adnp* sgRNA-targeted versus non-targeted Th2 cells. All shown pathways were enriched (p < 0.05). (I and J) (I) *Gata3* and (J) *Mik67* gene expression (from RNA-seq analysis) in *Adnp* sgRNA-targeted and non-targeted cells. Mean ± SD; unpaired two-sided t test; not significant (ns). See also Tables S1 and S2.

To identify additional regulators of *Il13* gene expression in mouse Th2 cells from IL-13-reporter mice (*Rosa26*^Cas9EGFP^*Il13*^tdTomato^), we undertook an unbiased retrovirus-mediated CRISPR-Cas9 screening approach using a sgRNA library targeting 1,131 TFs. Naive primary splenic CD4^+^ T cells were retrovirally transduced and differentiated into Th2 cells by initiating T cell receptor and IL-4 signaling pathways.^23^ After 3 days in the CRISPR culture conditions, transduced cells were sorted by IL-13Tom expression (BFP^+^IL-13Tom^+^ and BFP^+^IL-13Tom^−^) and purified for next-generation sequencing (NGS) of integrated sgRNA inserts. Consistent with the known roles of GATA3 and STAT6 in Th2 cell differentiation, sgRNAs targeting *Gata3* and *Stat6* were highly enriched in IL-13Tom^−^ populations (Figure 1A; Table S1) as were *Yy1*, *Nfkb1*, *Junb*, and *Batf*. Notably, *Adnp* was identified as a previously unappreciated candidate for the regulation of IL-13 expression (Figure 1A).

There is little-to-no characterization of ADNP in the context of immune cells. Deletion of the *Adnp* in mice leads to compromised brain formation and embryonic lethality,^24^ and mutations in *ADNP* underlie the complex neurological and developmental disorder ADNP syndrome (Helsmoortel-Van der Aa autism syndrome, *ADNP*-related disorder).^25^^,^^26^ ADNP is reported to be expressed ubiquitously and have pleiotropic roles including repressive functions during embryonic cell (EC) development.^27^^,^^28^^,^^29^ We found that *Adnp* gene and protein expression were equivalent in naive T, Th1, and Th2 cells (Figures 1B and 1C). ADNP contains 9 zinc-finger domains and a C-terminal homeobox domain mediating DNA binding, and an 8 amino acid neuroprotective peptide called NAP (NAPVSIPQ) which mediates neuroprotection when secreted.^30^ However, treatment with NAP had no effect on Th2 cell differentiation (Figure 1D).

To confirm ADNP as a regulator of IL-13 expression in Th2 cells we used sgRNAs to individually genetically ablate *Adnp in vitro*, compared with non-targeting and *Gata3* sgRNAs. *Adnp* disruption reduced the number of IL-13^+^ cells by approximately 50% (Figures 1E and 1F). We next performed bulk RNA-seq to compare gene expression in cultured Th2 cells following *Adnp* deletion as compared with non-deleted cells. Targeting *Adnp* resulted in the downregulation of 190 genes (including *Maf*, *Il4*, and *Flt3l*) and upregulation of 117 genes (Figure 1G; Table S2). Kyoto Encyclopedia of Genes and Genomes (KEGG) pathway analysis indicated that the differentially expressed genes (DEGs) downregulated in the absence of ADNP were associated with “Asthma” pathways, which correlates with the roles of IL-13 and Th2 cells in allergic asthma (Figure 1H). Importantly, the effect of targeting *Adnp* was independent of GATA3 expression, which was essential for IL-13 production (Figure 1I). Additionally, the expression of *Mki67* indicated that the proliferation of ADNP-deficient Th2 cells was not perturbed (Figure 1J). Thus, ADNP represents a previously unappreciated regulator of immune gene activation.

### ADNP-deficient mice have reduced antigen-specific Th2 cell responses

**Figure 2:**
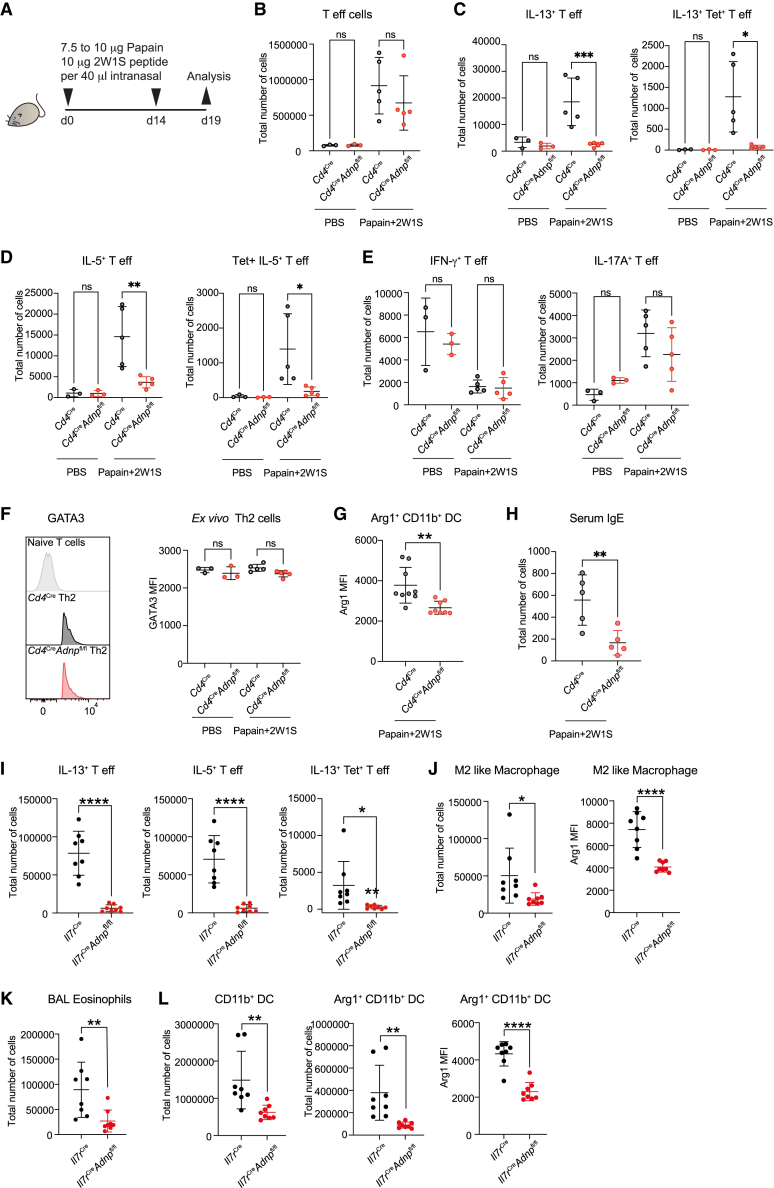
ADNP-deficient mice have reduced antigen-specific Th2 cell responses (A) Schematic of the experimental induction of type 2 inflammation in the mouse lung with papain and 2W1S peptide. (B–H) Flow cytometric analysis of lung (B) total Th effector cells, (C) total and 2W1S-tetramer-specific IL-13-producing Th effector cells, (D) total and 2W1S-tetramer-specific IL-5-producing Th effector cells, (E) total IFN-γ and IL-17A-producing Th effector cells, (F) GATA3 expression in Th2 cells, (G) Arg1 expression in Arg1^+^CD11b^+^ dendritic cells (DCs), and (H) serum IgE quantified by ELISA. Data are representative of 2 independent experiments; mean ± SD; one-way ANOVA with Tukey’s post-hoc test. ^∗∗∗^p < 0.001, ^∗∗^p < 0.01, ^∗^p < 0.05, not significant (ns). (I–L) Flow cytometric analysis of lung (I) total and 2W1S-tetramer-specific IL-13-producing Th effector cells and total IL-5-producing Th effector cells, (J) total M2 macrophage and Arg1 expression in M2 macrophage, (K) total bronchoalveolar eosinophils, (L) total CD11b^+^ and Arg1^+^CD11b^+^ DCs and Arg1 expression in Arg1^+^CD11b^+^ DCs. Data are representative of 2 independent experiments; mean ± SD; one-way ANOVA with Tukey’s post-hoc test. ^∗∗∗∗^p < 0.0001, ^∗∗^p < 0.01, ^∗^p < 0.05. See also Figures S1 and S2.

To investigate the potential impact of ADNP in primary cells and Th2 cell responses *in vivo*, we established *Adnp*^fl/fl^ mice intercrossed with *Cd4*^Cre^ mice in which ADNP was deleted preferentially in T cells (*Cd4*^Cre^*Adnp*^fl/fl^ mice, Figures S1A and S1B). To induce robust type 2-cell-mediated allergic immune responses in the lungs of these mice, we primed and re-challenged animals intranasally with the protease-allergen papain.^31^^,^^32^ The 2W1S peptide immunogen was co-administered with papain to track antigen-specific CD4^+^ T cells using the 2W1S:I-Ab MHCII tetramer^33^ (Figures 2A, S1C, and S1D). We found that although the total numbers of Th effector cells (viable CD45^+^CD3^+^CD4^+^CD44^+^ cells) were unchanged (Figure 2B), there was a reduction in IL-13 and IL-5 expression by total T effector cells and 2W1S peptide-specific T cells in the *Cd4*^Cre^*Adnp*^fl/fl^ mice as compared with controls in the lung (Figures 2C and 2D). In contrast, we observed no changes in IFN-γ^+^ or IL-17^+^ cells (Figure 2E). Numbers of IL-4^+^ Th effector cells in the lung and in the mediastinal lymph nodes (medLNs) were not impacted by ADNP deletion, though they were relatively rare (Figure S2A). The compromised IL-13 and IL-5 production was not associated with changes in GATA3 expression (Figure 2F). Total numbers of NK cells, B cells, natural killer T (NKT) cells, regulatory T (T reg) cells, CD4^+^ naive T cells and CD4^+^ Th cells were not altered at homeostasis or after antigen challenge in the lungs from *Cd4*^Cre^*Adnp*^fl/fl^ mice (Figures S2B and S2C). However, we observed a reduction in lung CD8^+^ T cells (Figure S2D). We analyzed the thymus of *Cd4*^Cre^*Adnp*^fl/fl^ mice to determine if there was a defect in the genesis of CD8^+^ T cells. We found comparable numbers of CD4 single-positive (SP) and CD8 SP cells (Figure S2E), but an increased number of CD4/CD8 double-positive (DP) cells and a decrease in NKT cells (Figures S2E and S2F). These results suggested that ADNP may play unappreciated roles at specific stages of lymphocyte development from DP to NKT cells, but not from DP to SP CD4 and CD8 cells. By performing combined adoptive transfer of equal numbers of *Cd4*^Cre^*Adnp*^fl/fl^ (CD45.2) and control bone marrow cells (CD45.1) into lethally irradiated recipient mice (expressing both CD45.1and CD45.2), we assessed the T cell-intrinsic versus extrinsic effects of ADNP deficiency (Figure S2G). Reconstitution was equivalent (Figures S2H and S2I). Co-reconstitution with wild-type T cells was sufficient to reverse the CD8^+^ T cell deficit observed above, indicating that it was not intrinsic to *Cd4*^Cre^*Adnp*^fl/fl^ T cells (Figure S2J). By contrast, the defects in IL-13, IL-5, and IL-4-producing Th effector cells were intrinsic to the *Cd4*^Cre^*Adnp*^fl/fl^ cells as they were not reversed by the presence of wild-type T cells (Figure S2K).

In the lungs of *Cd4*^Cre^*Adnp*^fl/fl^ mice, we did not detect major reductions in M2 macrophages or eosinophils that are dependent on IL-13 and IL-5, respectively, but did observe reduced arginase 1 (Arg1) expression by CD11b^+^ dendritic cells (DCs) (Figure 2G) and a reduction in serum IgE, which are dependent on IL-13 and IL-4 (Figure 2H). We speculated whether the residual type 2 immunity observed could result from the partial functional redundancy between Th2 cells and ILC2s, with ILC2 acting as additional ADNP-dependent sources of type 2 cytokine production in *Cd4*^Cre^*Adnp*^fl/fl^ mice (Figure S2L). Therefore, we intercrossed *Adnp*^fl/fl^ mice with *Il7ra*^Cre^ mice (*Il7ra*^Cre^*Adnp*^fl/fl^ mice) to delete ADNP in all lymphocytes. These mice were challenged as above with papain and 2W1S peptide. In *Il7ra*^Cre^*Adnp*^fl/fl^ mice very few effector Th and 2W1S peptide-specific T cells or ILC2 expressed IL-13 or IL-5 (Figures 2I and S2M). Deletion of *Adnp* in all lymphocytes resulted in a notable reduction in B cells and NK cells (Figure S2N), and a modest decrease in CD4^+^ and CD8^+^ T cells (Figure S2O), further suggesting that ADNP may have additional roles in lymphocyte development or proliferation. Allied to the impairment in lymphocyte-derived type 2 cytokine production in *Il7ra*^Cre^*Adnp*^fl/fl^ mice, we observed reduced M2-like macrophage polarization (Figure 2J) and eosinophilia (Figure 2K). In addition, ADNP-deficient mice presented notable reductions in CD11b^+^ and Arg1^+^CD11b^+^ DC recruitment and lower expression of Arg1 (Figure 2L), indicative of inefficient type 2 cytokine stimulation. Together, these results confirm a critical role for ADNP in Th2 cell polarization and ILC2 type 2 cytokine expression and type 2 immunity. Indeed, deletion of *Adnp* in all lymphocytes almost totally abrogated the type 2 immune response to lung allergen.

**Figure 3:**
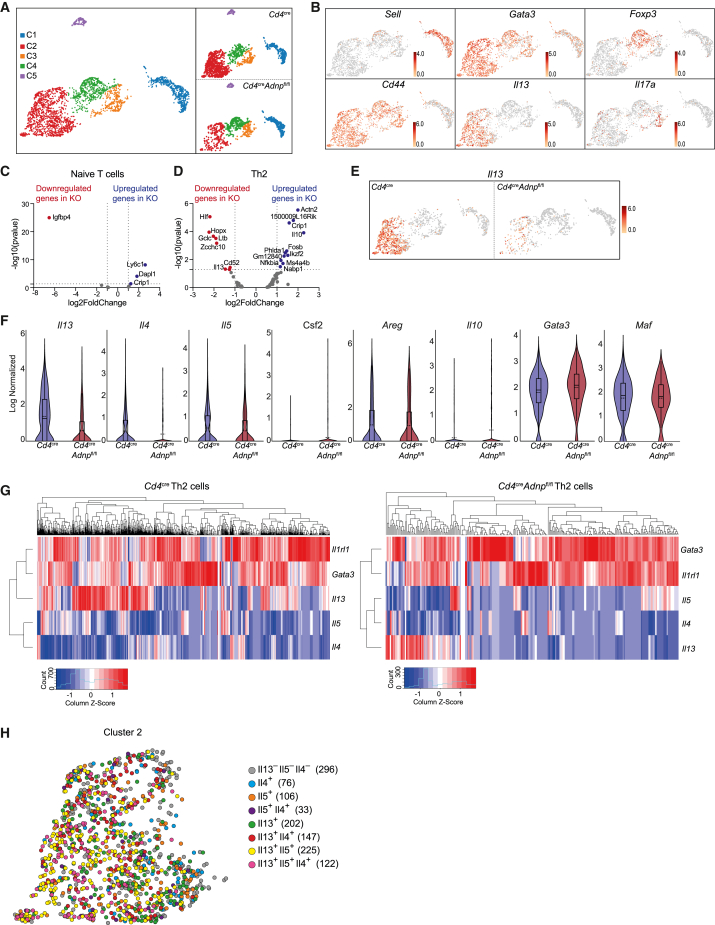
Single-cell gene expression analysis confirms the importance of ADNP for IL-13 expression (A) UMAP plot of single-cell gene expression analysis of naive CD4^+^CD44^−^CD62L^+^CD25^−^ T cells and CD4^+^CD44^+^CD62L^−^ST2^+^ effector T cells purified from the lungs of papain-challenged *Cd4*^Cre^ and *Cd4*^Cre^*Adnp*^fl/fl^ mice. (B) UMAP plot with expression (log_2_ expression) of indicated genes per individual cell. (C and D) Volcano plot comparing genes from naive T cells (cluster 1) (C) and Th2 cells (cluster 2) (D) from *Cd4*^Cre^ and *Cd4*^Cre^*Adnp*^fl/fl^ (KO) mice. (E) UMAP plot with expression (log_2_ expression) of *Il13* per individual cell. (F) Violin plots with expression (log_2_ expression) of indicated genes. Rectangle, solid line, and dashed line represent the interquartile range, the median and the mean, respectively. (G) Heatmap showing the expression of indicated genes in cells from cluster 2 from *Cd4*^Cre^ (left) and *Cd4*^Cre^*Adnp*^fl/fl^ (right) mice. Columns represent cluster 2 individual cells and rows represent the different genes. (H) UMAP plot showing cluster 2. Cells are colored by their cytokine expression pattern as shown, with number of cells indicated in parentheses. See also Figure S3.

To better understand how ADNP modulates Th2 cell responses to allergen we performed single-cell RNA sequencing (scRNA-seq) analysis of naive CD4^+^CD44^−^CD62L^+^CD25^−^ T cells and CD4^+^CD44^+^CD62L^−^ST2^+^ effector T cells purified from the lungs of papain-challenged *Cd4*^Cre^ and *Cd4*^Cre^*Adnp*^fl/fl^ mice (Figure 2A). The cells formed 5 distinct clusters in both control and ADNP-deficient mice (Figure 3A). Cells in cluster 1 expressed genes characteristic of naive T cells, e.g., CD62L (encoded by *Sell*) while lacking expression of *Cd44* (Figures 3B and S3A). Cluster 2 was characterized by the expression of Th2-cell-associated genes, including *Il13*, *Gata3*, and *Il1rl1* (encoding ST2) (Figures 3B and S3A). Cluster 3 represented a minor population expressing *Il17a* and *Il18r1*, typical Th17 cell-associated genes (and were probably purified due to background anti-ST2 antibody staining) (Figures 3B and S3A). Cells in cluster 4 expressed *Foxp3*, characteristic of Treg cells (Figures 3B and S3A). Cluster 5 was composed of mitotic cells characterized by expression of genes associated with cell proliferation and cell cycle regulation (*Mki67*, *Birc5*, and *Ccna2*) (Figure S3B) and was removed from further analysis for simplification.

Comparison of control naive T cells with ADNP-deficient naive T cells indicated only four DEGs (Figure 3C). The only downregulated gene was *Igfbp4*, which is associated with inhibition of cell proliferation and the stimulation of apoptosis but does not alter lymphocyte development.^34^ The absence of ADNP resulted in very few DEG in Treg or Th17 cells (Figure S3C). However, *Hopx* and *Hlf* gene expression was notable in all the Th cell populations analyzed, but not in naive T cells (Figures 3C, 3D, and S3C). Hopx is expressed in T cells where it can regulate IL-2 expression,^35^^,^^36^ which can promote T cell proliferation. *Hlf* has not been associated with T cell function. Comparing the DEG from control and ADNP-deficient Th2 cells demonstrated that *Il13* expression was reduced in the absence of ADNP (Figures 3D–3G) indicating that ADNP is required for the expression of *Il13* following the differentiation of naive T cells. *Il4* and *Il5* expression was not dysregulated, though cell numbers were small (Figures 3D, 3F, and 3G). Furthermore, the scRNA-seq data also confirmed previous reports^37^^,^^38^^,^^39^ that the expression of the *Il13*, *Il4*, and *Il5* are not always synchronized, with individual cells expressing one, two, or all these cytokines (Figures 3G and 3H). Additionally, *Il13* expression was more associated with *Gata3* and *Il1rl1* expression (Figure 3G), and this association was reduced in the absence of ADNP (Figure 3G). We also confirmed that *Gata3* expression was not affected by ADNP deletion (Figures 3F and 3G). Other type 2 cytokines such as GM-CSF (encoded by *Csf2*) and *Areg*, were not impacted by ADNP deficiency or were not sufficiently expressed to allow detection by scRNA-seq analysis (Figure 3F). *Il10* was one of the upregulated genes in *Cd4*^Cre^*Adnp*^fl/fl^ Th2 cells but was only detected in a few cells (Figure 3F). In contrast to what we observed *in vitro*, *Maf* expression was not reduced in ADNP-deficient Th2 cells (Figure 3F).

These data indicate that ADNP is required for *Il13* expression following the differentiation of naive T cells, even in the context of normal GATA3 expression.

### ADNP is associated with genes involved in lymphocyte differentiation

We next sought to identify the mechanism by which ADNP regulates IL-13 production and Th2 cell polarization. Studying ES cell differentiation, Buhler and colleagues reported that the CHD4, ADNP, HP1γ complex (called ChAHP) impairs chromatin accessibility around its DNA-binding site preventing access to transcriptional activators,^28^ in part by competing for CTCF sites and thus modifying TADs.^27^^,^^28^^,^^29^^,^^40^ Further, an ADNP complex with the chromatin remodeling regulators CHD4 and BRG1 is known to repress gene expression in mouse ES cells.^27^^,^^41^

**Figure 4:**
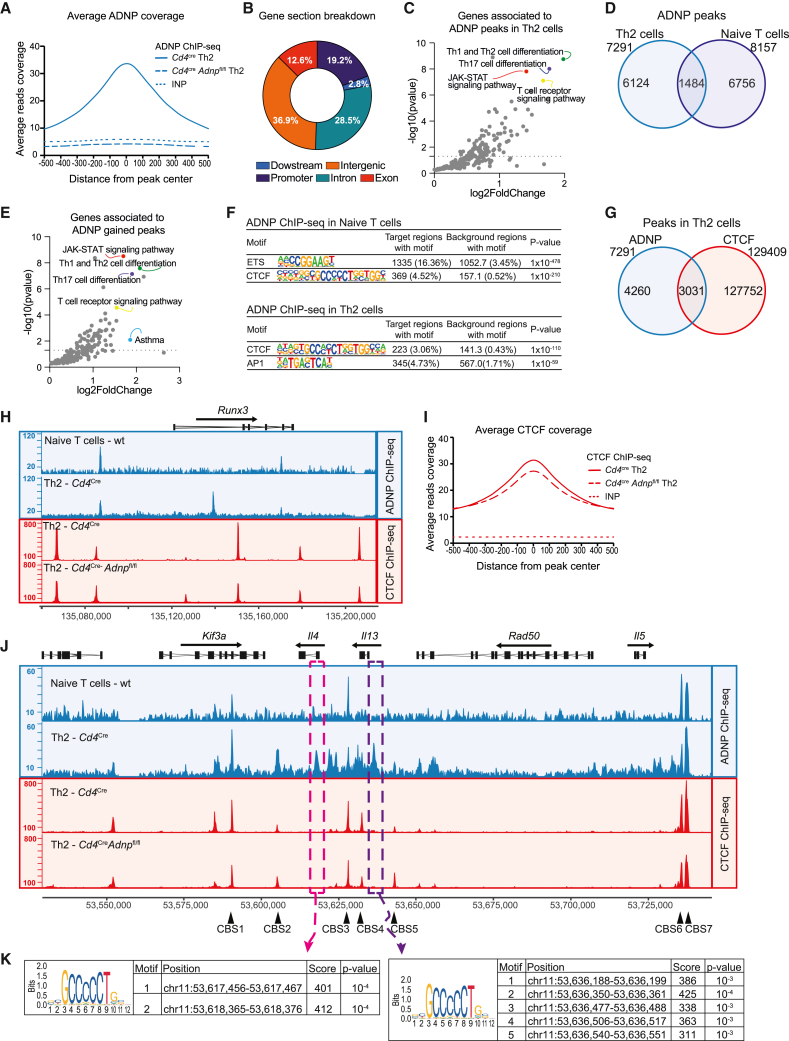
ADNP is associated with genes involved in lymphocyte differentiation (A) Average ChIP-seq signal over all ADNP peaks in Th2 cells. Data are representative of 3 biological replicates. (B) Pie chart displaying distribution of 7,291 ADNP peaks across genomic features. Promoter includes peaks between 1-kb upstream and 1-kb downstream of the transcription start site (TSS). Downstream includes peaks up to 1-kb downstream transcription termination site (TTS). (C) KEGG pathway analysis of the genes associated to ADNP peaks. All pathways shown were enriched (p < 0.05). (D) Venn diagram showing the overlap between ADNP ChIP-seq peaks in Th2 and naive T cells. Peak list was generated using two biological replicates. (E) KEGG pathway analysis of the genes associated with ADNP gained peaks (peaks present in Th2 cells but not in naive T cells). All pathways shown were enriched (p < 0.05). (F) Top two enriched motifs of ADNP peaks in naive T cells and Th2 cells. Enrichment was assessed using a one-sided cumulative binomial distribution in HOMER. (G) Venn diagram showing the overlap between ADNP and CTCF ChIP-seq peaks in Th2 cells. Peak list was generated using two biological replicates. (H) Representative binding profiles of ADNP in wild-type naive T cells and *Cd4*^Cre^ Th2 cells and CTCF in *Cd4*^Cre^ or *Cd4*^Cre^*Adnp*^fl/fl^ Th2 cells at the *Runx3* locus. Data are representative of 3 biological replicates. (I) Average ChIP-seq signal over all CTCF peaks in *Cd4*^Cre^ or *Cd4*^Cre^*Adnp*^fl/fl^ Th2 cells. Data are representative of 2 biological replicates. (J) Representative binding profiles of ADNP in wild-type naive T cells and *Cd4*^Cre^ Th2 cells; and CTCF in *Cd4*^Cre^ or *Cd4*^Cre^*Adnp*^fl/fl^ Th2 cells at the type 2 cytokine locus. Black arrows indicate CTCF-binding sites (CBSs). Data are representative of 3 biological replicates. (K) Predicted CTCF motifs within the ADNP ChIP-seq peaks at the *Il4* and *Il13* promoter regions (magenta and purple rectangles in J). Prediction was performed using Jaspar transcription factor database. See also Figure S4 and Tables S3 and S4.

We therefore sought to define the molecular interactions of ADNP in naive T cells and *in-vitro*-differentiated Th2 cells using chromatin immunoprecipitation followed by sequencing (ChIP-seq). The *in-vitro*-differentiated *Cd4*^Cre^*Adnp*^fl/fl^ Th2 cells showed reduced IL-13 expression (Figures S4A and S4B), similar to *in vivo* Th2 cells (Figure 2C). However, no change was observed in IL-5 expression in *in-vitro*-differentiated cells (in contrast to *in vivo*) (Figures S4A and S4B). IFN-γ expression by Th1 cells was also unaffected, and we observed no deficit in T cell proliferation as assessed by expression of Ki67 or the activation marker CD25 (Figure S4B). To validate the assay, we performed ChIP-seq using ADNP-deficient *Cd4*^Cre^*Adnp*^fl/fl^ Th2 cells to establish assay background (Figure 4A). As in ES cells,^28^ around 60% of ADNP-binding sites in Th2 cells were associated with protein-coding genes and almost 20% were located at promoters (Figure 4B). Analysis of enriched KEGG pathways revealed that ADNP target genes included those associated with Th2 cell differentiation, JAK-STAT, and T cell receptor signaling, and asthma-related pathways (Figure 4C; Tables S3 and S4). Only 20% of ADNP-binding sites in Th2 cells were already occupied by ADNP in naive T cells, indicating the dynamic regulation of ADNP binding during Th2 cell differentiation (Figure 4D). By comparing ADNP binding in naive T cells and Th2 cells, we observed that those sites newly occupied by ADNP in differentiated T cells were enriched for “Th1 and Th2 cell differentiation” pathways including the genes encoding IL-4, IL-13, and cMaf (Figure 4E), whereas those originally present in the naive cells were not (Figure S4C).

Motif enrichment analysis of ADNP-associated peaks from naive T cells identified the CTCF and ETS motifs, whereas ADNP was associated with CTCF and AP-1 sites in Th2 cells (Figure 4F). This raised the possibility that in some locations ADNP might bind directly to sites in the vicinity of AP-1 sites, or directly to the AP-1 motif, or that it may associate with other proteins, such as AP-1 factors to interact with chromatin indirectly or in combination. Notably, we had identified the *Junb* and *Batf* genes, which encode the AP-1 factors JUNB and BATF, in our CRISPR-Cas9 screen (Figure 1A). The association of ADNP with CTCF motifs was confirmed using anti-CTCF ChIP-seq, with around 40% of ADNP-binding sites overlapping with CTCF binding (Figure 4G), similar to previously published ES cell data.^29^ However, in Th2 cells in contrast to ES cells, ADNP and CTCF did not appear to compete for the CTCF-binding sites, since CTCF binding was not affected by the absence of ADNP (Figures 4H and 4I). Moreover, the ADNP peaks identified in the naive T cells showed a greater association with CTCF than the ADNP peaks identified in the Th2 cells (Figure S4D). This suggested that an alternative mechanism may underlie ADNP-mediated gene regulation in Th2 cells.

At the Th2 cell cytokine locus, confirming previous reports, we found that CTCF bound predominantly to CTCF-binding sites (CBSs) 1, CBS3, CBS6, and CBS7^20^ (Figure 4J). Super-imposition with ADNP binding showed that ADNP colocalized with CTCF at CBS1, CBS6, and CBS7 (Figure 4J), and that ADNP was already present at these locations in naive T cells. However, we did not observe that the deletion of ADNP influenced CTCF binding at these loci either (Figure 4J). Unexpectedly, we observed that ADNP bound to the *Il13* (purple rectangle) and *Il4* (magenta rectangle) promoter regions. ADNP was recruited to these locations during Th2 cell differentiation and was not present in naive T cells (Figure 4J). These regions contain predicted CTCF-binding motifs, which were not bound by CTCF in T cells, providing a platform for ADNP interaction (Figure 4K). The *Il5* promoter region was not bound by ADNP (Figure 4J).

Together, these results demonstrate that under Th2 cell differentiation conditions ADNP binds to the type 2 cytokine locus in a region known to regulate *Il13* gene expression, which includes CTCF and AP-1 motifs.

### ADNP associates with chromatin remodeling factors and AP-1 factors in Th2 cells

**Figure 5:**
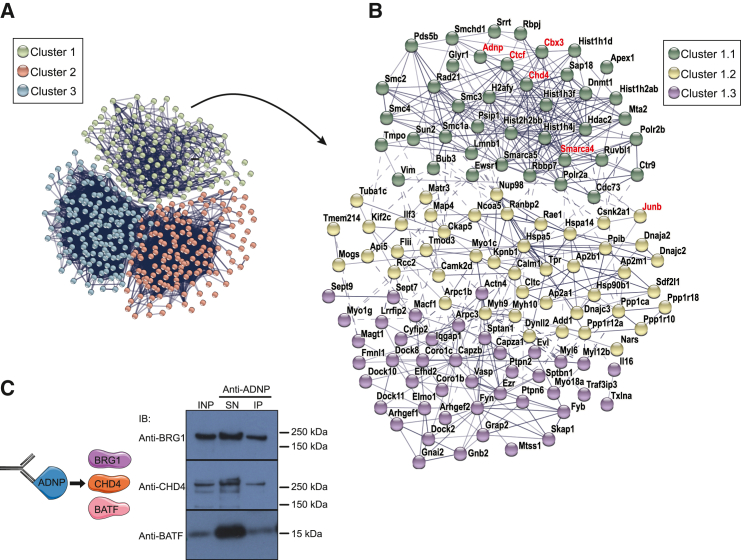
ADNP associates with chromatin remodeling factors and AP-1 factors in Th2 cells (A) STRING analysis of the 457 proteins immunoprecipitated with anti-ADNP from Th2 cells and identified by mass spectrometry. Connecting lines between proteins denote the confidence of the interactions (line thickness indicates the strength of data support). K means clustering was performed to identify three clusters represented by individual colors. Co-expression and co-occurrence were removed from active interaction sources. (B) STRING analysis of cluster 1 in (A). (C) Protein associations confirmed by co-immunoprecipitation experiments. Detection of BRG1 (181 kDa), CHD4 (226 kDa), and BATF (14 kDa) proteins in immunocomplexes generated with Th2 cell nuclear lysate co-immunoprecipitated with anti-ADNP antibody. INP, input nuclear extract; SN, supernatant; IP, immunoprecipitation elution. Data are representative of 2 independent experiments. IB refers to immunoblotting antibody. See also Figure S5 and Table S5.

Next, to investigate possible ADNP-binding partners in Th2 cells, we performed anti-ADNP immunoprecipitation followed by mass spectrometry (MS) analysis. We identified 457 proteins as compared with isotype control (Table S5). STRING protein interaction analysis (https://string-db.org/) showed the complexity of ADNP interactions in Th2 cells with 3 major clusters: cluster 1 chromatin-binding (GO:0003682) and TF-binding (GO:0008134) factors; cluster 2, RNA binding (GO:0003723), and cluster 3, mRNA binding molecular functions (GO:0003729) (Figure 5A). We focused on cluster 1 which contains ADNP and can be subdivided into 3 subclusters (Figure 5B). Cluster 1.1 includes ADNP, histones, and components of the chromatin remodeling complexes (NuRD, SWI/SNF, and ChAHP complexes), for example, CHD4, HP1γ (CBX3), and BRG1 (SMARCA4), which associate with ADNP in ESC.^28^^,^^29^ CTCF and cohesins were also present in this cluster. Gene ontology analysis indicated enrichment of chromosome organization (GO:0051276), chromatin organization (GO:0006325), regulation of gene expression (GO:0010468), and histone modification (GO:0016570) biological processes. Of note, cluster 1.2 contained the AP-1 TF JUNB suggesting an association with ADNP (Figure 5B).

We performed co-immunoprecipitation (coIP) experiments to verify ADNP interaction partners. We confirmed that endogenous ADNP co-immunoprecipitated with CHD4 and BRG1 (Figure 5C). Anti-BRG1 immunoprecipitation also pulled down ADNP and CHD4 (Figure S5A). Although ADNP did not directly co-precipitate JUNB (Figure S5B), it did co-precipitate BATF (Figure 5C), which binds to JUNB to form a dimeric AP-1 TF. Further, endogenous BATF pulled down ADNP, BRG1, and JUNB (Figure S5C). These data demonstrate that ADNP can interact with CHD4 and BRG1 as well as the pioneer TF AP-1, composed of JUNB and BATF. Both BATF and JUNB are known to be important for Th2 cell differentiation and regulation of the Th2 cytokine locus.^12^^,^^42^

### ADNP is required for efficient recruitment of CHD4 and BRG1 to the Th2 cytokine locus

**Figure 6:**
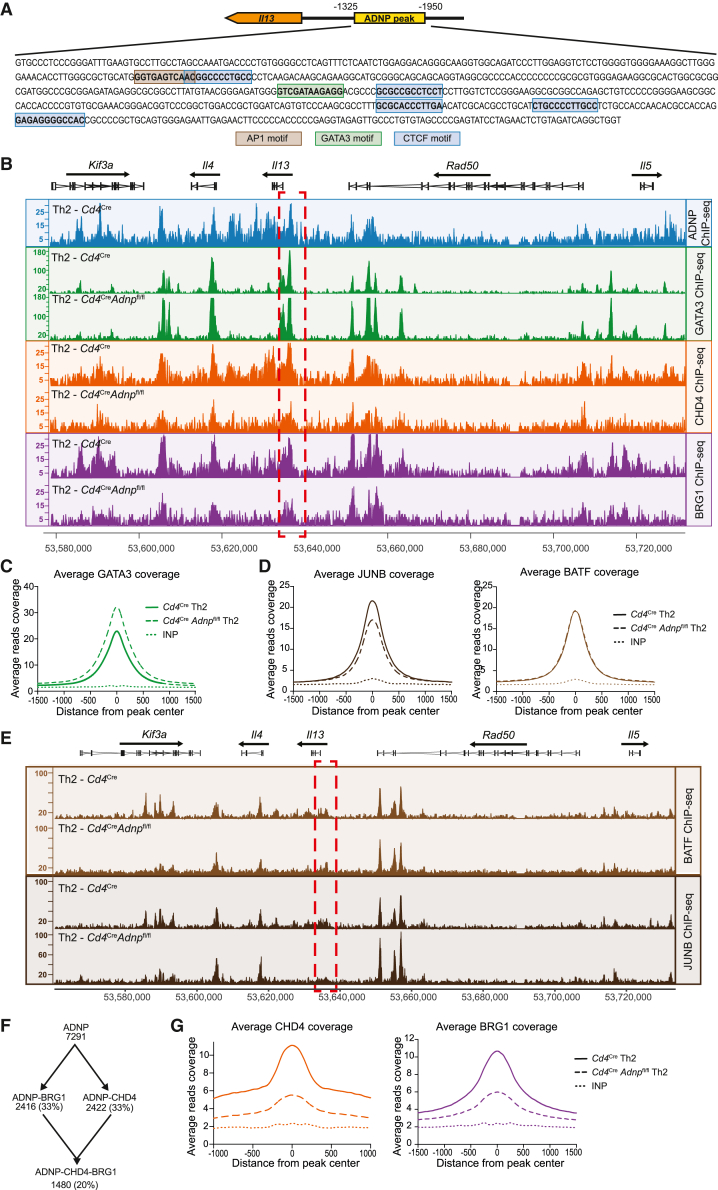
ADNP is required for efficient recruitment of CHD4 and BRG1 to the Th2 cytokine locus (A) Predicted AP-1, GATA3, and CTCF motifs within ADNP ChIP-seq peak upstream of *Il13* promoter. Prediction was performed using Jaspar transcription factor database. (B) Representative binding profiles of ADNP, GATA3, CHD4, and BRG1 in Th2 cells from *Cd4*^Cre^ or *Cd4*^Cre^*Adnp*^fl/fl^ mice at the type 2 cytokine locus. Data are representative of 3 biological replicates. (C) Average ChIP-seq signal over all GATA3 peaks in *Cd4*^Cre^ or *Cd4*^Cre^*Adnp*^fl/fl^ Th2 cells. Data are representative of 2 biological replicates. (D) Average ChIP-seq signal over all JUNB and BATF peaks in *Cd4*^Cre^ or *Cd4*^Cre^*Adnp*^fl/fl^ Th2 cells. Data are representative of 3 biological replicates. (E) Representative binding profiles of BATF and JUNB in Th2 cells from *Cd4*^Cre^ or *Cd4*^Cre^*Adnp*^fl/fl^ mice at the type 2 cytokine locus. Data are representative of 2 biological replicates. (F) Number and percentages of all ADNP, ADNP-BRG1, ADNP-CHD4, and ADNP-CHD4-BRG1 overlapping ChIP-seq peaks in Th2 cells. Peak list was generated using two biological replicates. (G) Average ChIP-seq signal over all CHD4 and BRG1 peaks in *Cd4*^Cre^ or *Cd4*^Cre^*Adnp*^fl/fl^ Th2 cells. Data are representative of 3 biological replicates. See also Figure S6.

Given the interaction of ADNP with CHD4, BRG1, and BATF, we performed ChIP-seq to investigate their localization within the Th2 locus. As indicated by anti-ADNP ChIP-seq analysis, ADNP binds upstream of the *Il13* promoter in a region harboring one AP-1 motif followed by a GATA3 motif^12^^,^^43^ and five predicted CTCF motifs (Figure 6A). Using anti-GATA3 ChIP-seq, we confirmed that this region aligns with the conserved GATA3 response element (CGRE) (Figure 6B), which has been postulated to regulate Th2 cell cytokine production via GATA3 binding.^43^ We determined that GATA3 binding at the CGRE preceded ADNP binding, since GATA3 was present at this location in naive T cells, before ADNP recruitment (Figures 4J and S6A). We observed a modest increase of GATA3 binding in the CGRE region and across the genome of ADNP-deficient Th2 cells (Figures 6B and 6C), which may arise from the ADNP-deficient Th effector cells becoming arrested and unable to progress beyond the GATA3-binding stage. Anti-JUNB and anti-BATF ChIP-seq indicated that JUNB and BATF binding also occurred independently of ADNP (Figures 6D and 6E), suggesting that they, similar to GATA3, can access and bind DNA before or without prior binding of ADNP. However, although ADNP co-precipitated with BATF (and BATF co-precipitated JUNB), it is currently unclear whether this AP-1 complex binds to the AP-1 site present in the CGRE where AP-1 enrichment was only modest (Figure 6E).^12^

Anti-BRG1 and anti-CHD4 ChIP-seq demonstrated that these chromatin remodeling proteins also colocalized with ADNP binding at the CGRE (Figure 6B), with ∼20% of ADNP-binding sites in Th2 cells coincident with BRG1 and CHD4 binding (Figure 6F), but not HP1γ binding (described as a repressor in the ChAHP complex in ES cells^28^) (Figure S6B). In the absence of ADNP, we observed a strong reduction in the binding of CHD4 and BRG1 to the CGRE, and at other ADNP-binding regions across the genome (Figure 6G), indicating a critical role for ADNP in recruiting these chromatin modifiers. ADNP also colocalizes with GATA3, BRG1, and CHD4 at the *Il4* promoter region where there was a reduction in BRG1 and CHD4 binding in ADNP-deficient cells (Figure 6B).

These data demonstrate that ADNP is not required for the binding of the pioneer factors GATA3 and AP-1 to the Th2 locus but is necessary for recruitment of BRG1 and CHD4 to chromatin.

### The absence of ADNP at the *Il13* promoter reduces local H3K27 acetylation and DNA accessibility

**Figure 7:**
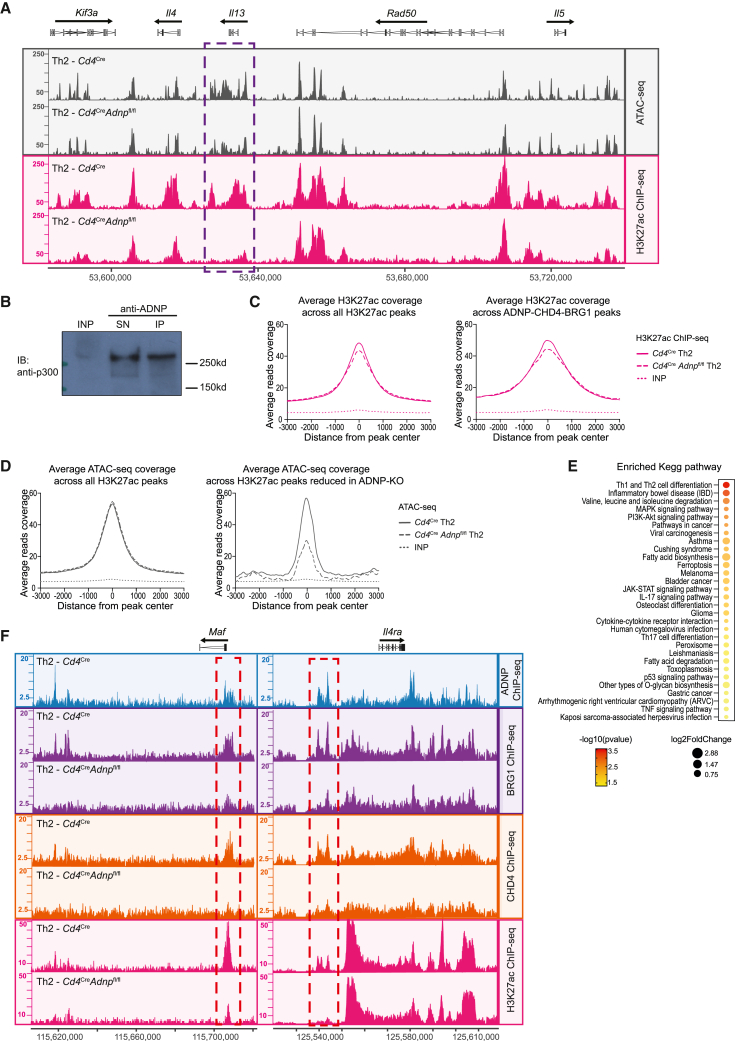
Absence of ADNP at the *Il13* promoter reduces local H3K27 acetylation and DNA accessibility (A) Representative ATAC-seq and H3K27ac ChIP-seq tracks from Th2 cells from *Cd4*^Cre^ or *Cd4*^cre^*Adnp*^fl/fl^ mice in the type 2 cytokine locus. Data are representative of 2 biological replicates. (B) Detection of p300 protein (218 kDa) in immunocomplex generated with Th2 cell nuclear lysate co-immunoprecipitated with anti-ADNP antibody. INP, input nuclear extract; SN, supernatant; IP, immunoprecipitation elution. Data are representative of 2 independent experiments. IB refers to immunoblotting antibody. (C) Average ChIP-seq signal over all H3K27ac peaks or over ADNP-CHD4-BRG1 peaks in *Cd4*^Cre^ or *Cd4*^Cre^*Adnp*^fl/fl^ Th2 cells. Data are representative of 3 biological replicates. (D) Average ATAC-seq signal over all H3K27ac peaks or over H3K27ac peaks that were reduced in *Cd4*^Cre^*Adnp*^fl/fl^ Th2 cells. Data are representative of 2 biological replicates. (E) KEGG pathway analysis of genes associated to the genomic regions where H23K27ac was reduced in *Cd4*^Cre^*Adnp*^fl/fl^ Th2 cells. All shown pathways were enriched (p < 0.05). (F) Representative binding profiles of ADNP, CHD4, BRG1, and H3K27ac in Th2 cells of *Cd4*^Cre^ or *Cd4*^Cre^*Adnp*^fl/fl^ mice at the *Maf* and *Il4ra* loci. Data are representative of 2 biological replicates. See also Figure S7 and Table S6.

Next, we investigated how ADNP, with CHD4 and BRG1, altered gene expression. We found using assay for transposase-accessible chromatin sequencing (ATAC-seq) assays that without ADNP full accessibility to the *Il13* locus was not initiated (Figure 7A). To investigate this defect in accessibility, we assessed the acetylation status of the *Il13* locus in the presence and absence of ADNP. Histone H3 lysine 27 acetylation (H3K27ac) is known to shape active promoters and enhancers by opening chromatin, thereby allowing the transcriptional machinery to assemble.^44^^,^^45^ Indeed, the *Il13* locus in Th2 cells was marked by H3K27ac (Figure 7A, purple rectangle). We found that the absence of ADNP led to a profound localized deficit in H3K27 acetylation at the *Il13* locus (Figure 7A, purple rectangle). Since CHD4 is important for the recruitment of the HAT P300 to the Th2 cytokine locus, including to the CGRE,^46^ we assessed the relationship of P300 with ADNP and found that P300 also associated with ADNP in Th2 cells (Figure 7B). Although we identified co-localization of ADNP, GATA3, BRG1, and CHD4 at the *Il4* promoter, similar to the *Il13* promoter, we did not observe a pronounced or reproducible change in gene accessibility or H3K27 acetylation at this locus in the absence of ADNP (Figure 7B). As the proportion of Th2 cells that express IL-4 is relatively small when compared with IL-13-expressing cells (Figures 3F–3H and S2A), it is likely that these IL-4-positive cells become diluted within whole-population analysis such as ATAC-seq and ChIP-seq, making it difficult to determine the modulation of this locus.

The co-localization of ADNP-CHD4-BRG1 was highly associated with H3K27ac regions (Figure 7C). As expected, regions where H3K27 acetylation was reduced in ADNP-deficient Th2 cells displayed a reduction in DNA accessibility (Figure 7D). Pathway enrichment analyses of the genes associated with those regions revealed Th cell differentiation, JAK-STAT, and asthma, among the enriched pathways (Figure 7E). Comparing those genes with the genes associated to ADNP-CHD4-BRG1 peaks, we found 228 genes that were present in both lists (Table S6), and 8 of those were related to the Th1 and Th2 cell differentiation pathway. In addition to the *Il13* locus, the *Il4ra* and *Maf* loci also feature convergent binding of the chromatin modifiers and a coincident reduction in H3K27 acetylation in ADNP-deficient Th2 cells (Figure 7F). Notably, the *Maf* and *Il4ra* genes play key roles in Th2 differentiation.^47^^,^^48^^,^^49^

These results support a key role for ADNP in focusing Th2 gene expression patterns to potentiate Th2 cell differentiation and activate type 2 cytokine production, acting as a critical component in the recruitment of chromatin remodeling proteins CHD4, BRG1, and P300.

## Discussion

Our CRISPR-Cas9 screen identified ADNP as a factor, which has not been characterized previously in immune cells, as well as several TFs already known to play roles in Th2 cell differentiation and type 2 cytokine production. Conditional deletion of ADNP from T cells, or all lymphocytes, revealed that ADNP was required for efficient type 2 immune responses to allergic challenge and also in lymphocyte development. Mechanistically, ADNP bound to unoccupied CTCF motifs within the CGRE of the type 2 cytokine locus upstream of the *Il13* coding region and was critical for localized histone acetylation and gene accessibility, leading to IL-13 expression. ADNP performs this function by recruiting a complex of chromatin remodeling factors including BRG1, CHD4, and P300 to gene regulatory regions.

The situation at the *Il4* and *Il5* loci appears to be more complex. At the *Il4* locus ADNP colocalizes with GATA3, BRG1, and CHD4 indicating that an ADNP-dependent mechanism may be directly involved in *Il4* regulation and *in vitro* Th2 cell differentiation assays indicated a reliance upon ADNP for *Il4* expression, which was shown to be T cell intrinsic in the context of *in vivo* bone marrow transfer and antigen challenge. However, we observed inconsistency in ADNP-dependent IL-4 expression in some *in vivo* experiments, potentially due to the relatively small numbers of IL-4-positive cells. By contrast, we could not identify direct ADNP binding at the *Il5* locus, despite the expression of IL-5 being impaired in a Th2 cell-intrinsic ADNP-dependent manner following allergen challenge. This suggests that *Il5* gene regulation may not be due to direct ADNP binding to the *Il5* gene, but that ADNP influences *Il5* transcription by an alternative mechanism, perhaps by contributing to dysregulated 3D chromatin architecture.^37^^,^^50^ The prominent role for ADNP in regulating IL-13 expression was further illustrated by single-cell gene expression analysis of Th2 cells following allergen challenge which showed that *Il13* expression was predominant within a mix of *Il4*, *Il5*, and *Il13* expressing cells (representing single, double, and triple-positive Th2 cells), and that these were almost undetectable in the absence of ADNP. These data support previous observations indicating that the cytokine genes in this cluster can be independently regulated by discrete control mechanisms and argue for a role for ADNP in this process.^38^^,^^39^

Mutations in the gene encoding ADNP underlie ADNP syndrome, which is characterized by neurological and developmental abnormalities.^25^^,^^51^ More recent studies have focused on the ability of ADNP to act genome-wide to locally restrict gene expression in ES cells to prevent spontaneous cell differentiation.^27^^,^^28^^,^^29^^,^^40^ This effect results from ADNP associating with CHD4 and the transcriptional repressor HP1γ to form ChAHP complexes, which locally restrict chromatin accessibility by competing for CBSs and thereby modifying TADs.^27^^,^^28^^,^^29^^,^^40^ A similar role for ADNP in gene repression in ES cells was reported by Sun and colleagues, who found ADNP associated with CHD4 and BRG1.^27^^,^^28^^,^^29^^,^^40^^,^^41^ In contrast to these repressive roles for ADNP in ES cells, our data from primary Th2 cells demonstrated that ADNP can also play a fundamental and previously unappreciated role in directly promoting gene expression. This suggested a mechanism distinct from the repression model previously proposed in ES cells.^27^^,^^28^^,^^29^^,^^40^ Indeed, when we analyzed the DNA binding of CTCF and ADNP in primary wild-type or ADNP-deficient Th2 cells, we found no evidence that ADNP competed for CBSs within the type 2 cytokine locus, or across the genome. Instead, we determined that unoccupied CTCF motifs in the *Il13* promoter were bound by ADNP during the differentiation of naive T cells to cytokine-producing Th2 cells.

The deletion of ADNP in T cells resulted in a pronounced defect in the recruitment of the histone-modifying factors CHD4 and BRG1 to the CGRE upstream of the *Il13* promoter, and coIP verified that in Th2 cells ADNP associated with CDH4, BRG1, and P300. Although CHD4, which induces ATP-dependent distortion of nucleosomal DNA during chromatin remodeling^52^ is commonly viewed as a component of the repressive NuRD complex,^53^ it can also act as a transcriptional activator. Indeed, CHD4 is required for *Cd4* gene transcription in T cells through its association with the HAT P300 at the *Cd4* enhancer.^54^ A similar association was reported in Th2 cells where CHD4 was found to bind to GATA3, and was proposed to recruit P300 to the CGRE.^46^ The amino-terminal region of CHD4 has also been demonstrated to associate with BRG1 to activate transcription,^55^ which in turn has also been reported to bind to the *Il13* promoter in Th2 cells.^56^ Thus, although an ADNP-CHD4-BRG1 complex can be repressive in ES cells,^27^ our results are congruent with BRG1 hydrolyzing ATP to drive chromatin accessibility and transcription at the type 2 gene cluster in T cells.^57^

In ES cells ADNP maintains multipotency with an overarching requirement to suppress cell differentiation programs. In this situation, the competition between ADNP and CTCF helps control TAD formation and repression predominates.^29^ In Th2 cells, ADNP is predominantly associated with activation during the polarization of cytokine production in response to cytokine and T cell receptor signaling. Although the mechanism underlying these differences is not clear, it is possible that the interactions that we observed between ADNP and the pioneer factors AP-1 and GATA3 may help focus ADNP to active loci in T cells in response to IL-4 signaling. GATA3 is essential for Th2 development^2^^,^^58^^,^^59^ and AP-1 factors (BATF and JUNB) also play key roles in this cellular program and cytokine expression.^12^^,^^13^^,^^42^^,^^60^^,^^61^ Our results indicate that GATA3 and AP-1 bind to the CGRE independently of ADNP but that ADNP appears to form a complex that includes BATF as well as BRG1, CHD4, and P300. In addition, BATF binds JUNB, and CHD4 and JUNB have been reported to bind GATA3.^46^ This suggests that a larger complex can form at genomic locations where there is a conjunction of GATA3, AP-1, and ADNP binding to their juxtaposed DNA motifs to facilitate the ADNP-dependent recruitment of BRG1, CHD4, and P300 and provide specific locus activation. Indeed, when we looked beyond the type 2 gene cluster for loci with GATA3, AP-1, ADNP, BRG1, and CHD4 protein co-localization, which also showed a deficit in acetylation and histone accessibility in ADNP-deficient *in-vitro*-cultured Th2 cells, we found that these loci encoded MAF and the IL-4 receptor alpha (IL-4Rα). The MAF proto-oncogene was one of the first TFs reported to activate the *Il4* promoter, leading to IL-4 expression and Th2 cell differentiation.^47^ Furthermore, IL-4Rα is the primary binding chain for IL-4, which stimulates Th2 cell differentiation through the activation of STAT6 and the induction of GATA3 expression.^2^^,^^49^ These results suggest that ADNP (with associated factors) may be capable of promoting a Th2 cell feedback loop that reinforces the Th2 cell phenotype. However, we did not observe a reduction in MAF expression *in vivo*, suggesting that there are alternative ADNP-independent pathways that can regulate MAF expression *in vivo*. By contrast, our data indicate no such ADNP-independent regulation of the *Il13 locus*.

The critical biological role of this ADNP bridge is vividly revealed by the incapacity of mice with ADNP-deficient T cells or ADNP-deficient lymphocytes to respond efficiently to allergen challenge. These mice displayed impaired production of type 2 cytokines by Th2 and ILC2 and failed to mount a robust type 2 immune response. Although ADNP has not been identified as a common factor in asthma susceptibility, unlike IL-4, IL-5, and IL-13, it was reported among a subset of genes that were differentially expressed by CD4^+^ lymphocytes and that predicted more atopic from less atopic children.^62^ However, there are currently no reports of children with ADNP syndrome presenting specific immunological disorders, though recurring infections (∼50% of cases) are reported.^26^

Together, our results are consistent with ADNP functioning as an adapter or bridge to specifically localize CHD4 and BRG1 to ADNP-bound CTCF motifs forming a complex that can include the acetylase P300 and drive histone acetylation and genome accessibility. This ADNP-dependent mechanism is essential to promote Th2 cell differentiation and type 2 cytokine production in response to IL-4 signaling. Our results also raise the possibility that ADNP may play additional key roles in hematopoiesis and immunity.

### Limitations of the study

For the future, manipulation of the CTCF and/or ADNP motifs within the whole cytokine locus and in the vicinity of individual cytokine genes will help to define ADNP-binding specificity. This could be attempted in primary cells or gene-modified mice to allow *in vitro* and *in vivo* readouts of ADNP function. Furthermore, the application of single-cell ChIP-seq and ATAC-seq could also provide improved resolution of the chromatin changes which are dependent on ADNP. Moreover, our study raised the involvement of ADNP in pan-lymphocyte development within the bone marrow, and of NKT cell differentiation in the thymus, and additional studies will be required to identify the mechanisms of action of ADNP in these processes. In addition, it will be important to assess the relevance of ADNP on human Th2 cell function, as our study focused on the mouse model.

## STAR★Methods

### Key resources table

**Table:** 

REAGENT or RESOURCE	SOURCE	IDENTIFIER
**Antibodies**		

Anti-mouse CD3e (145-2C11) BV510	BioLegend	Cat#100353; RRID: AB_2565879
Anti-mouse CD4 (GK1.5) PerCP/Cy5.5	BioLegend	Cat#100434; RRID: AB_893324
Anti-mouse CD8a (53-6.7) Alexa Fluor700	BioLegend	Cat#100730; RRID: AB_493703
Anti-mouse/human CD11b (M1/70) PE-Cy7	BioLegend	Cat#101216; RRID: AB_312799
Anti-mouse CD19 (6D5) Alexa Fluor700	BioLegend	Cat#115528; RRID: AB_493735
Anti-mouse CD25 (PC61) PE	BioLegend	Cat#102008; RRID: AB_312857
Anti-mouse CD25 (PC61) BV510	BioLegend	Cat#102042; RRID: AB_2562270
Anti-mouse/human CD44 (IM7) PerCP/Cy5.5	BioLegend	Cat#103032; RRID: AB_2076204
Anti-mouse/human CD44 (IM7) BV605	BioLegend	Cat#103047; RRID: AB_2562451
Anti-mouse CD62L (MEL-14) BV421	BioLegend	Cat#104436; RRID: AB_2562560
Anti-mouse CD127 (SB/199) biotin	BioLegend	Cat#121104; RRID: AB_493502
Anti-mouse F4/80 (BM8) BV785	BioLegend	Cat#123141; RRID: AB_2563667
Anti-mouse FceR1 (MAR-1) Alexa Fluor700	BioLegend	Cat#134324; RRID: AB_2566734
Anti-mouse/human IL-5 (TRFK5) APC	BioLegend	Cat#504306; RRID: AB_315330
Anti-mouse IFN-γ (XMG1.2) BV785	BioLegend	Cat#505838; RRID: AB_2629667
Anti-mouse Arginase 1 (A1exF5) PE	eBioscience	Cat#12-3697-82; RRID: AB_2734839
Anti-mouse CD3 (17A2) Alexa Fluor700	eBioscience	Cat#56-0032-82; RRID: AB_529507
Anti-mouse CD4 (GK1.5) ef450	eBioscience	Cat#48-0041-82; RRID: AB_10718983
Anti-mouse CD4 (GK1.5) Alexa Fluor700	eBioscience	Cat#56-0041-82; RRID: AB_493999
Anti-mouse CD4 (GK1.5) FITC	eBioscience	Cat#11-0041-82; RRID: AB_464892
Anti-mouse CD8a (53-6.7) PE-Cy5	eBioscience	Cat#15-0081-82; RRID: AB_468706
Anti-mouse CD11b (M1/70) Alexa Fluor700	eBioscience	Cat#56-0112-82; RRID: AB_657585
Anti-mouse CD11c (N418) Alexa Fluor700	eBioscience	Cat#56-0114-82; RRID: AB_493992
Anti-mouse/human CD44 (IM7) APC	eBioscience	Cat#17-0441-82; RRID: AB_469390
Anti-mouse CD45.1 (A20) PE-Cy5	eBioscience	Cat#15-0453-82; RRID: AB_468759
Anti-mouse CD45.2 (104) Alexa Fluor700	eBioscience	Cat#56-0454-82; RRID: AB_657752
Anti-mouse/human Gata-3 (TWAJ) eFluor660	eBioscience	Cat#50-9966-42; RRID: AB_10596663
Anti-mouse GR-1/Ly-6G/C (RB6-8C5) Alexa Fluor700	eBioscience	Cat#56-5931-82; RRID: AB_494007
Anti-mouse IL-13 (eBio13A) PE	eBioscience	Cat#12-7133-82; RRID: AB_763559
Anti-mouse IL-13 (eBio13A) PE-Cy7	eBioscience	Cat#25-7133-82; RRID: AB_2573530
Anti-mouse Ki67 (SolA15) PE-Cy7	eBioscience	Cat#25-5698-82; RRID: AB_11220070
Anti-mouse KLRG1 (2F1) PerCP-eFluor710	eBioscience	Cat#46-5893-82; RRID: AB_10670282
Anti-mouse Ly-6G (1A8-Ly6G) PerCP-eFluor710	eBioscience	Cat#46-9668-82; RRID: AB_2573893
Anti-mouse MHCII (M5/114.15.2) eFluor450	eBioscience	Cat#48-5321-82; RRID: AB_1272204
Anti-mouse NK1.1 (PK136) Alexa Fluor700	eBioscience	Cat#56-5941-82; RRID: AB_2574505
Anti-mouse TCR beta (H57-597) eFluor450	eBioscience	Cat#48-5961-82; RRID: AB_11039532
Anti-mouse TER-119 (TER-119) Alexa Fluor700	eBioscience	Cat#56-5921-82; RRID: AB_2815252
Fixable Viability Dye eFluor 780	eBioscience	Cat#65-0865-18
Anti-mouse CD45 (30-F11) BUV395	BD Biosciences	Cat#564279; RRID: AB_2651134
Anti-mouse NK1.1 (PK136) BUV395	BD Biosciences	Cat#564144; RRID: AB_2738618
Anti-mouse SiglecF (E50-2440) Alexa Fluor647	BD Biosciences	Cat#562680; RRID: AB_2687570
Streptavidin BUV737	BD Biosciences	Cat#612775; RRID: AB_2870104
Anti-mouse ST2 (DJ8) FITC	MD bioproducts	Cat#101001F; RRID: AB_947549
2W1S-tetramer PE	NIH Tetramer Facility	N/A
anti-ADNP	Novus Biologicals	Cat#NBP1-89236; RRID: AB_11008573
anti-CTCF	Cell Signaling	Cat#2899; RRID: AB_2086794
anti-GATA3	Cell Signaling	Cat#5852; RRID: AB_10835690
anti-CHD4	Cell Signaling	Cat#12011; RRID: AB_2734702
anti-JUNB	Cell Signaling	Cat#3753; RRID: AB_2130002
anti-H3K27ac	Cell Signaling	Cat#8173; RRID: AB_10949503
anti-BRG1	abcam	Cat#ab110641; RRID: AB_10861578
anti-BATF	abcam	Cat#ab236876;
anti-HP1g	abcam	Cat#ab217999; RRID: AB_217999
anti-p300	abcam	Cat#ab275378
anti-CD3	2B Scientific	Cat#Ab00105-1.1
anti-CD28	2B Scientific	Cat#AGEL0759
anti-IFNg	2B Scientific	Cat#AGEL2200
anti-IL-4	BioLegend	Cat#504101

**Chemicals, peptides, and recombinant proteins**		

2W1S peptide	Designer Bioscience	N/A
papain	Sigma-Aldrich	Cat#76216
rmIL-2, carrier-free	Biolegend	Cat#575406
rmIL-4, carrier-free	Biolegend	Cat#574306
rmIL-12, carrier-free	Biolegend	Cat#577006
PMSF	Sigma Aldrich	Cat#P7626
collagenase I	Gibco	Cat#17100017
DNase I, from bovine pancreas	Sigma-Aldrich	Cat#D5025/DN25
Percoll	GE Healthcare	Cat#17-0891-01
cOmplete protease inhibitor	Roche	Cat#4693116001
Pierce 660nm protein assay reagent	ThermoFisher,	Cat#22660
A/G dynabeads)	Thermo Scientific	Cat#88802
1X NuPage LDS sample buffer	Invitrogen	Cat#NP0008
ECL western blotting detection reagent	GE Healthcare	Cat#RPN2106
protein A Dynabeads	ThermoFisher	Cat#10002D
NAP	Biotechne	Cat#6779

**Critical commercial assays**		

Mouse IgE Uncoated ELISA kit	Invitrogen	Cat#88-504460-88
Ovation RNA-seq System V2	Tecan	Cat#7102-32
Ovation Ultralow Library Systems	Tecan	Cat#0344NB-32
Fixable Dye eFluor 780	Invitrogen	Cat#65-0865-14
BD Cytofix/Cytoperm Plus reagents	BD Biosciences	Cat#555028
Protein Transport Inhibitor Cocktail	eBioscience	Cat#00-4980-9
Gibson assembly	New England BioLabs	Cat#E5510S
NEB T4 DNA ligase	New England BioLabs	Cat#M0202S
RPMI 1640 + GlutaMAX	GIBCO	Cat#61870-010
Fetal Calf Serum	GIBCO	Cat#10270-106
2-mercaptoethanol	Sigma-Aldrich	Cat#M6250
DMEM	GIBCO	Cat#10564011
Fugene HD Transfection Reagent	Promega	Cat#E2311
OPTI-MEM	GIBCO	Cat#31985062
DNeasy Blood & Tissue Kits	QIAGEN	Cat#*69504*
Herculase II Fusion DNA polymerase	Agilent	Cat#600675
KAPA library quantification kit	Roche	Cat#KK4824
RNeasy Plus Micro kit	Qiagen	Cat#74034
truChIP Chromatin Shearing kit	Covaris	Cat#520154
Foxp3 Staining kit reagents	eBioscience	Cat#00-5523-00
Qiagen MinElute kit	Qiagen	Cat#28004
Kappa HiFi HotStart Ready mix	Roche	Cat#KK2601
Illumina Tagment DNA Enzyme and Buffer	Illumina	Cat#20034197
SPRI Ampure XP beads	Beckman Coulter	Cat#A63881

**Deposited data**		

scRNAseq	This paper	GEO: GSE218017
ATACseq	This paper	GEO: GSE218017
ChIPseq	This paper	GEO: GSE218017

**Experimental models: Cell lines**		

Platinum-E retroviral packaging cells	Cell biolabs	Ca#RV-101

**Experimental models: Organisms/strains**		

Mouse: *Rosa26*^Cas9EGFP^	The Jackson Laboratory	JAX 026179
Mouse: *Il13*^tdTom^	Barlow et al.^38^	N/A
Mouse: *Il7r*^Cre^	Schlenner et al.^63^	MGI:4441349
Mouse: *CD4*^Cre^	Taconic	Ca#4196
Mouse: *Adnp*^tm1a(KOMP)Wtsi^	KOMP repository	RRID:MMRRC_051854-UCD
Mouse: C57BL/6JOla	Jackson Labs (Bred in LMB)	Cat#000664; RRID: IMSR_JAX:000664

**Oligonucleotides**		

Primer for sgRNA-insert amplification (Forward) AATGGACTATCATATG CTTACCGTAACTTGAAAGTATTTCG	Sigma-Aldrich	N/A
Primer for sgRNA-insert amplification (Reverse) CTTTAGTTTGTATGTCTGTT GCTATTATGTCTACTATTCTTTCC	Sigma-Aldrich	N/A

**Software and algorithms**		

FlowJo	FlowJo, LLC	RRID: SCR_008520
Prism 9	GraphPad Prism	RRID: SCR_002798
Cutadapt	N/A	https://journal.embnet.org/index.php/embnetjournal/article/view/200
Trim Galore	Babraham Bioinformatics	https://www.bioinformatics.babraham.ac.uk/projects/trim_galore/
DESeq2	Love et al.^64^	https://bioconductor.org/packages/release/bioc/vignettes/DESeq2/inst/doc/DESeq2.html
10x Cell Ranger	10x Genomics	https://support.10xgenomics.com/single-cell-gene-expression/software/pipelines/latest/installation
10x Genomics Loupe Browser	10x Genomics	https://www.10xgenomics.com/products/loupe-browser
Scaffold programme (Proteome Software Inc., USA)^65^	Keller et al.^65^	Proteome Software Inc., USA
Bowtie2 (version 2.3.5.1)	Langmead et al.^66^	https://bowtie-bio.sourceforge.net/bowtie2/manual.shtml
HOMER	Heinz et al.^67^	http://homer.ucsd.edu/homer/index.html
SeqMonk software (v1.48.0)	Babraham Bioinformatics	https://www.bioinformatics.babraham.ac.uk/projects/seqmonk/
FACSDiva software	BD Biosciences	https://www.bdbiosciences.com/en-gb/products/software/instrument-software/bd-facsdiva-software
Macs2 (v2.1.2)	Zhang et al.^68^	https://github.com/macs3-project/MACS/wiki
STAR (version 2.6.0a)	N/A	https://github.com/alexdobin/STAR

**Other**		

Mouse Brie CRISPR knockout pooled library	Addgene	Ca#73633
MSCV-pU6-(BbsI)-CcdB-(BbsI)-Pgk-Puro-T2A-BFP	Addgene	Ca#86457

### Resource availability

#### Lead contact

Further information and requests for resources and reagents should be directed to and will be fulfilled by the lead contact, Andrew McKenzie (anm@mrc-lmb.cam.ac.uk).

#### Materials availability

This study did not generate new unique reagents.

### Experimental model and subject details

#### Mice

*Rosa26*^Cas9EGFP^ (JAX 026179),^69^ *Il13*^tdTom^,^38^ *Il7r*^Cr^^e^ ^63^ and *CD4*^Cre^ (Taconic, model #4196) mice were on the C57BL/6 background. C57BL/6 controls were bred in-house. *Adnp*^flox^ targeted ES cells (to delete exon 5) were purchased from KOMP repository (*Adnp*^tm1a(KOMP)Wtsi^) and *Adnp*^fl^ mouse line was generated by methods previously described.^70^ All mice were maintained in the Medical Research Council ARES animal facility under specific pathogen-free conditions, at 19-23^o^C with a 12-h light-dark cycle. In individual experiments, mice were matched for age, sex and background strain and all experiments undertaken in this study were done so with the approval of the LMB Animal Welfare and Ethical Review Body (AWERB) and of the UK Home Office.

### Method details

#### Antibodies

Antibodies against the following proteins were used in immunoprecipitation and ChIP-seq experiments: ADNP (Novus Biologicals NBP1-89236), CTCF (Cell Signaling, #2899), GATA3 (Cell Signaling, #5852), CHD4 (Cell Signaling, #12011), BRG1 (abcam, ab110641), JUNB (Cell Signaling, #3753), BATF (abcam, ab236876), H3K27ac (Cell Signaling, #8173), HP1g (abcam, ab217999). In flow cytometry experiments we used antibodies from BioLegend (CD3e (BV510, 145-2C11, 1:300 dilution), CD4 (ef450, GK1.5, 1:500 dilution) or (BV785, RM-4-5, 1:500 dilution), CD8a (Alexa Fluor700, BV421 or BV785, 53-6.7, 1:500 dilution), CD11b (PE-Cy7, M1/70, 1:1000 dilution), CD19 (Alexa Fluor700 or BV605, 6D5, 1:500 dilution), CD25 (PE or BV510, PC61, 1:300 dilution), CD44 (PerCP/Cy5.5 or BV605, IM7, 1:500 dilution), CD45 (BV510, 30-F11, 1:500 dilution), CD62L (BV421, MEL-14, 1:500 dilution), CD127 (biotin, SB/199, 1:500 dilution), F4/80 (BV785, BM8, 1:500 dilution), FceR1 (Alexa Fluor700, MAR-1, 1:500 dilution), IL-5 (APC, TRFK5, 1:300 dilution), IFN-g (BV785, XMG1.2, 1:300 dilution), eBioscience (Arginase 1 (PE, A1exF5, 1:300 dilution), CD3e (Alexa Fluor 700, 17A2, 1:300 dilution), CD4 (Alexa Fluor700 or FITC, GK1.5, 1:500 dilution), CD8a (FITC or PE-Cy7, 53-6.7, 1:500 dilution), CD11b (Alexa Fluor700, M1/70, 1:500 dilution), CD11c (Alexa Fluor 700 or PE-Cy7, N418, 1:500 dilution), CD19 (PerCP-Cy5.5 or PE-Cy7, eBio1D3, 1:500 dilution), CD44 (FITC or APC, IM7, 1:500 dilution), CD45 (FITC, 30-F11, 1:500 dilution), FceR1 (PE-Cy7, MAR-1, 1:500 dilution), Gata-3 (eFluor 660, TWAJ, 1:300 dilution), GR-1/Ly-6G/C (Alexa Fluor700 or PE-Cy7, RB6-8C5, 1:500 dilution), IL-13 (PE or PE-Cy7, eBio13A, 1:300 dilution), KLRG1 (PerCP-eFluor710, 2F1, 1:500 dilution), Ly-6G (PerCP-eFluor710, 1A8-Ly6G, 1:500 dilution), MHCII (eFluor450, M5/114.15.2, 1:1000 dilution), NK1.1 (Alexa Fluor700 or PE-Cy7, PK136, 1:500 dilution), TER-119 (Alexa Fluor700 or PE-Cy7, TER-119, 1:500 dilution)), BD Biosciences (CD45 (BUV395, 30-F11, 1:500 dilution), NK1.1 (BUV395, PK136, 1:300 dilution), SiglecF (Alexa Fluor 647, E50-2440, 1:500 dilution), Streptavidin (BUV737, 1:500 dilution)), MD bioproducts (ST2 (FITC, DJ8, 1:500 dilution)) and the NIH Tetramer Facility (2W1S-tetramer, PE, 1:500 dilution). ‘Lineage’ staining included antibodies specific for CD3, CD4, CD8, CD11b, CD11c, CD19, FceRI, GR-1, NK1.1 and TER-119. All samples were co-stained with a cell viability dye (Fixable Dye eFluor 780, Invitrogen).

#### Adoptive transfers

Bone marrow cells were purified by flow cytometry from wildtype control mice (CD45.1) and from *Cd4*^Cre^*Adnp*^fl/fl^ (CD45.2). Cells from both sources were mixed at a ratio of 1:1 and implanted via tail vein injection into lethally irradiated (600 rad) CD45.1/CD45.2 recipients. After 6 weeks of cell transfer, mice were challenged with papain or PBS.

#### *In vivo* stimulation

Mice were anesthetized by isoflurane inhalation followed by the intranasal injection of papain (7.5 mg for males, 5 mg for females, Sigma-Aldrich #76216) with or without 2W1S peptide (50 mg, Designer Bioscience) in 40 μl PBS on days 0 and 14. Mice were sacrificed for analysis on day 19.

#### Tissue preparation

Cell suspensions from spleen, lymph nodes, and thymus tissue were obtained by passing the tissues through a 70-mm strainer. Lung tissue was predigested with 750 U ml^−1^ collagenase I (Gibco) and 0.3 mg ml^−1^ DNaseI (Sigma-Aldrich) and cell suspensions were obtained by passing the tissues through a 70-mm strainer. Red blood cells were removed by incubating with RBC lysis solution (140 mM NH_4_Cl, 17 mM Tris, pH 7.2). Lung lymphocytes were further enriched by centrifugation in 30% Percoll at 800*g* (GE Healthcare). Serum samples were obtaied following blood coagulation and centrifugation of coagulated cells at 3,300 x *g*. Serum IgE was quantified using the Invitrogen Mouse IgE Uncoated ELISA kit (88-504460-88) following the manufacturer’s instructions.

#### Flow cytometry

Single-cell suspensions were incubated with fluorochrome- or biotin-conjugated antibodies in the presence of anti-CD16/CD32 (Fc block, clone 2.4G2) as indicated. All samples were co-stained with a cell viability dye (Fixable dye eFluor780, Invitrogen). For cell sorting an iCyt Synergy (70-μm nozzle, Sony Biotechnology) was used. Intracellular cytokine staining was performed using BD Cytofix/Cytoperm Plus reagents (BD Biosciences) following pre-culture with RPMI, supplemented with 50 ng ml^-1^ phorbol 12-myristate 13-acetate (PMA), 500 ng ml^-1^ ionomycin and Protein Transport Inhibitor Cocktail (eBioscience), for 4 h at 37°C. Intracellular TF staining was performed using Foxp3 Staining kit reagents (eBioscience). Analysis was performed on an LSRFortessa system (BD Biosciences) with FACSDiva software (version 6.2, BD Biosciences). For cell sorting, an iCyt Synergy system (70-μm nozzle, Sony Biotechnology) was used. Data were analyzed with FlowJo software (version 10).

#### sgRNA cloning into retroviral expression vector

MSCV-pU6-(BbsI)-CcdB-(BbsI)-Pgk-Puro-T2A-BFP was a gift from Ralf Kuehn (Addgene plasmid # 86457; http://n2t.net/addgene:86457; RRID:Addgene_86457).^71^ Mouse Brie CRISPR pooled library was a gift from David Root and John Doench (Addgene #73633).^72^ Custom sgRNA libraries were synthesised by Twist Bioscience. sgRNA libraries were cloned into the retroviral vector by Gibson assembly. sgRNA library representation was verified by next generation sequencing to contain > 90% perfectly matching sgRNAs, < 0.5% undetected sgRNAs and a skew ratio of less than 10. sgRNA oligo pairs were purchased from Sigma-Aldrich. Individual CRISPR sequences were inserted into the retroviral vector by ligation (NEB T4 DNA ligase). Sequences of individual sgRNA-expressing constructs were confirmed by Sanger sequencing.

#### Th2 cell culture for CRISPR screening

Splenic naïve CD4^+^ T cells were sorted as Live CD4^+^CD44^lo^CD62L^hi^CD25^–^ cells. Cells were maintained in RPMI1640, 10% FCS with penicillin-streptomycin and 2-mercaptoethanol. Naïve CD4^+^ T cells were isolated from *Rosa26*^Cas9EGFP^ x *Il13*^tdTom^ mice and cultured on anti-CD3 coated plates (2B Scientific, 145-2C11, 5 mg ml^-1^, 37^o^C, 1 h), supplemented with anti-CD28 (2B Scientific, 37.51, 2 mg ml^-1^) and IL-2 (10 ng ml^-1^) for 24 hr. Cells were collected and mixed with retroviruses and spinoculated on retronectin-coated plates (Takara, 4 mg cm^-2^, non-TC-treated plate) at 37^o^C for 1 h. Cells were incubated further for 3 h at 37^o^C before transfer to fresh TC-treated plates until day 6. Fresh media containing 10 ng ml^-1^ IL-2 was supplemented at day 3. On day 6, cells were transferred to anti-CD3 coated plates and cultured in the presence of anti-CD28 (2 mg ml^-1^), IL-2 (10 ng ml^-1^), IL-4 (10 ng ml^-1^) and anti-IFNγ neutralising antibody (2B Scientific, 1 mg ml^-1^). After 3 days of differentiation, GFP^+^ BFP^+^ cells were sorted into IL13Tom^+^ and IL13Tom^-^ populations.

#### Retroviral production

Platinum-E retroviral packaging cells (Cell biolabs, #RV-101) were maintained in DMEM, 10% FCS with penicillin-streptomycin, supplemented with puromycin (1 mg ml^-1^) and blasticidin (10 mg ml^-1^). On the day before transfection, 3 million cells were seeded in a 100 mm culture dish in 10 ml of media without antibiotics. Cells were transfected at 70% confluency using Fugene HD Transfection Reagent (Promega). For each 100 mm culture dish, 950 ml OPTI-MEM (GIBCO) was mixed with 11 mg pCl-Eco, 22 mg library plasmid and 99 ml Fugene HD. The transfection mixture was incubated for 10 min at room temperature prior to addition. At 18 h post-transfection, the media was replaced with 10 ml fresh media, and viral supernatant was harvested at 48 and 72 h post-transfection. Cells were removed by filtering through a 0.45 mm syringe filter.

#### Genomic extraction and sequencing library preparation

Genomic DNA from sorted cells were extracted using the QIAGEN DNeasy Blood & Tissue Kits following the manufacturer’s protocol, with the exception of DNA elution in water instead of buffer AE. sgRNA-insert was first PCR-amplified using Herculase II Fusion DNA polymerase (Agilent) with primers (Forward) AATGGACTATCATATGCTTACCGTAACTTGAAAGTATTTCG and (Reverse) CTTTAGTTTGTATGTCTGTTGCTATTATGTCTACTATTCTTTCC, using up to 2 mg genomic DNA per 50 μl reaction. Equal volumes from each reaction were pooled and used for a further PCR amplification step to attach Illumina sequencing adaptors and Illumina P7 barcodes, using Herculase II Fusion DNA polymerase. The 330 bp library was gel purified and quantified using KAPA library quantification kit (Roche). Libraries were pooled and sequenced with a HiSeq 4000 at the CRUK Cambridge NGS facility.

#### Analysis of CRISPR screen results

20 nt sgRNA sequences were trimmed from backbone sequences using Cutadapt (version 1.4.1) (5’ GACGAAACACCG, 3’ GTTTTAGAGCTA). sgRNA sequences were aligned to reference sgRNA libraries using Bowtie2 (version 1.2.3). sgRNAs with counts less than 20 (genome-wide screens) or 50 (all other screens) in either of the populations were excluded from the analysis. The stat.wilcox function from the caRpools package (version 0.83) was applied to each screen separately. The function was modified to return the non-adjusted p-values. The stat.wilcox function collapses the sgRNAs to genes returning an enrichment score and a p-value for each gene. NT sgRNAs were used as a reference population. To combine data from screen replicates, the mean of enrichment score for each gene was calculated, and Fisher’s method was used to combine the p-values.

#### *In vitro* mouse Th cell culture

Splenic naïve T cells were sorted (CD4^+^CD44^-^CD25^-^CD62L^+^) and were cultured (250,000 cells /well) on anti-CD3 coated plates (5 mg ml^-1^), supplemented with anti-CD28 (2 mg ml^-1^) and IL-2 (10 ng ml^-1^). The following cytokines and neutralising antibodies were additionally supplemented in different Th conditions. Th1: IL-12 (10 ng ml^-1^) and anti-IL-4 neutralising antibody (BioLegend, 11B11, 1 mg ml^-1^). Th2: IL-4 (10 ng ml^-1^) and anti-IFNγ neutralising antibody (1 mg ml^-1^). Cells were passaged on day 2 or day 3, then analysed by flow cytometry on day 5. Where appropriate, 100 mM of the neuroprotective peptide called NAP (NAPVSIPQ) was supplemented during the *in vitro* differentiation.

#### RNA-sequencing

Cells were sorted by flow cytometry into PBS, 50% FCS, and RNA was extracted using the RNeasy Plus Micro kit (Qiagen). After assessment using a Bioanalyzer (Agilent), RNA was processed for RNA-seq using an Ovation RNA-seq System V2 (Nugen), fragmented using the Covaris M220 ultrasonicator and bar-coded using Ovation Ultralow Library Systems (Nugen). Samples were sequenced using an Illumina HiSeq 4000, by running a single-read 50-bp protocol (Cancer Research UK Cambridge Institute). Sequence data were trimmed to remove adaptors and sequences with a quality score below 30 using Trim Galore (version 0.50, Babraham Bioinformatics) and then aligned to the mouse genome (GRCm38) using STAR (version 2.6.0a), and differential expression was calculated using DESeq2 (version 1.18.1).^64^

#### Single-cell RNA sequencing

10x single-cell library preparation was performed using the 10x Genomics technology platform. The 10x Genomics Chromium Single Cell 3′ v3 protocol was followed to obtain 3′ libraries for subsequent sequencing. The reads were aligned to the mouse transcriptome (GRCm38), and expression was calculated using the 10x Cell Ranger (version 3.0.2) wrapper for the STAR aligner (version 2.60a). Separate libraries were generated using cells purified from the lungs of papain-challenged *Cd4*^Cre^ and *Cd4*^Cre^*Adnp*^fl/fl^ mice (7,500 CD4^+^CD44^+^CD62L^–^ST2^+^ effector T cells and 500 naïve CD4^+^CD44^–^CD62L^+^CD25^–^ T cells) and then combined using Cell Ranger. Analysis and statistical calculations were performed using the 10x Genomics Loupe Browser (https://support.10xgenomics.com/single-cell-gene-expression/software/visualization/latest/what-is-loupe-cell-browser).

#### Immunoprecipitation

Th2 cells were lysed in lysis buffer (50 mM Tris pH 8.0, 0.1% NP40, 10% glycerol and 2 mM EDTA), supplemented with 1x cOmplete protease inhibitor (Roche) and PMSF (Sigma Aldrich). After 10 min incubation on ice with intermittent mixing the lysates were centrifuged at 1,700 *g* at 4^o^C for 5 min and the supernatant was collected. The pelleted nuclei were resuspended in nuclear extraction buffer (50 mM Tris pH 8.0, 1 mM EDTA, 150 mM NaCl, 1% NP40 and 5% glycerol) supplemented with protease inhibitor cocktail and PMSF, and incubated on ice for 1 hour. Nuclear extract was collected by centrifugation at 13,000 x *g* at 4^o^C for 10 min. Protein concentration was quantified using the Pierce 660nm protein assay reagent (ThermoFisher, #22660). Lysates were incubated with antibodies (2 mg antibody per 100 mg protein) overnight at 4^o^C on a rotator. Immunocomplexes were precipitated with protein A/G dynabeads (Thermo Scientific #88802), washed three times with lysis buffer and once with TE buffer (10 mM Tris and 0.1 mM EDTA, pH 8). For western blot analysis, cell lysates or immunocomplexes were denatured by boiling at 95^o^C for 5 min in 1X NuPage LDS sample buffer (#NP0008) supplemented with 1% 2-mercaptoethanol. Proteins were resolved with Novex Tris-Glycine gels and transferred to PVDF membranes. Membranes were sequentially blocked with 5% BSA in PBST, incubated with primary and HRP-conjugated secondary antibodies and ECL western blotting detection reagent (GE Healthcare #RPN2106). For mass spectrometry analysis, the immunocomplexes were resuspended in 50mM NH_4_HCO_3_ followed by reduction with 10 mM DTT and alkylation with 55mM iodoacetamide. Then, proteins were digested (50 mM (NH₄)HCO₃ pH 8.0, 1μg trypsin, overnight, 37°C). Digestion was terminated by the addition of formic acid to a final concentration of 2% v/v. After separation (C18 Acclaim PepMap100 3 μm, 75 μm x 150 mm nanoViper, ThermoScientific Dionex, San Jose, USA), peptides were eluted with a gradient of acetonitrile. The analytical column outlet was directly interfaced via a modified nano-flow electrospray ionisation source, with a hybrid dual pressure linear ion trap mass spectrometer (Orbitrap Velos, ThermoScientific, San Jose, USA). Data dependent analysis was carried out, using a resolution of 30,000 for the full MS spectrum, followed by ten MS/MS spectra in the linear ion trap. MS spectra were collected over a m/z range of 300–2000. MS/MS scans were collected using a threshold energy of 35 for collision induced dissociation. LC-MS/MS data were then searched against a protein database (UniProt KB) using the Mascot search engine programme (Matrix Science, UK).^73^ Database search parameters were set with a precursor tolerance of 5 ppm and a fragment ion mass tolerance of 0.8 Da. Two missed enzyme cleavages were allowed and variable modifications for oxidized methionine, carbamidomethyl cysteine, pyroglutamic acid, phosphorylated serine, threonine and tyrosine were included. MS/MS data were validated using the Scaffold programme (Proteome Software Inc., USA).^65^ All data were additionally interrogated manually.

#### ChIP-seq using ChIPmentation

Chromatin extracts from *in vitro* cultured Th2 cells (1.0 × 10^7^ cells) were prepared using the truChIP Chromatin Shearing kit (Covaris), with 5 min of crosslinking and optimized shearing conditions (peak power, 75; duty factor, 10.0; cycles per burst, 200; duration, 300 s), to obtain fragments of ∼500 bp. Extracts were exposed to 1% SDS and diluted 10x with dilution buffer (5.5 mM EDTA, 55 mM Tris-HCl, pH 8, 200 mM NaCl, 0.5% NP-40). Chromatin extracts were incubated overnight at 4 °C with 2 μg of antibody. In addition, 25 μl protein A Dynabeads (Thermo Fisher Scientific) per immunoprecipitation were blocked in PBS containing 0.1% BSA (Sigma) by incubating overnight at 4 °C. The next day, beads were added to the chromatin extracts, followed by incubating for 1 h at 4 °C. Beads were collected and washed twice with low-salt buffer (0.1% SDS, 1% Triton X-100, 1 mM EDTA, 10 mM Tris-HCl, pH 8, 140 mM NaCl, 0.1% sodium deoxycholate), twice with high-salt buffer (0.1% SDS, 1% Triton X-100, 1 mM EDTA, 10 mM Tris-HCl, pH 8, 500 mM NaCl, 0.1% sodium deoxycholate), twice with LiCl buffer (10 mM Tris-HCl, pH 8, 1 mM EDTA, 250 mM LiCl, 0.5% NP-40, 0.5% sodium deoxycholate) and once with 10 mM Tris-HCl, pH 8. Chromatin–antibody–bead complexes were then subjected to tagmentation, followed by the elution of DNA, and libraries were amplified and purified as described previously.^74^ Pooled libraries were sequenced using an Illumina Novaseq 6000, running a pair-read 150-bp protocol (Cancer Research UK Cambridge Institute). Sequenced reads were aligned to the mouse genome (GRCm38) using Bowtie2 (version 2.3.5.1) with default parameters, and reads that could not be uniquely mapped were removed from further analyses. Aligned reads were visualised using the SeqMonk software (v1.48.0). HOMER^66^ (v4.10.4) software was used for motif find analysis. Peak calling analysis was performed using Macs2 (v2.1.2) and the target genes were defined by the closest gene from each peak (bedtools closest). Only target genes identified in two independent experiments were used in further analysis.

#### ATAC-seq

ATAC-seq was performed as previously described.^67^ 20,000 to 50,000 FACS purified cells were lysed using cold lysis buffer (10 mM Tris-HCl, pH 7.4, 10 mM NaCl, 3 mM MgCl_2_ and 0.1% NP-40) to obtain nuclei extract. Nuclei were immediately used in the transposase reaction (25 μl 2× TD buffer, 2.5 μl transposase (Illumina) and 22.5 μl nuclease- free water) for 30 min at 37 °C, followed by sample purification (Qiagen MinElute kit). Then, we amplified library fragments using Kappa HiFi HotStart Ready mix and 1.25 M of custom Nextera PCR primers as previously described.^68^ Libraries were purified using dual (0.5x-0.7x) SPRI Ampure XP beads (Beckman Coulter), pooled and were subjected to high-throughput sequencing. ATAC-seq data was aligned to the genome using the same pipeline as the ChIP-seq data.

### Quantification and statistical analysis

Statistical analysis was performed using GraphPad Prism version 9 software. Statistical significance was calculated by unpaired Student’s t-test (two-tailed), one-way or two-way ANOVA. ^∗∗∗∗^: P<0.0001, ^∗∗∗^: P<0.001, ^∗∗^: P<0.01, ^∗^: P<0.05, ns: not significant. No samples were excluded from the analysis.

## Data Availability

• Single-cell RNAseq, ATACseq and ChIPseq data are deposited with the National Center for Biotechnology Information Gene Expression Omnibus (GEO) under the accession number GSE218017. • This paper does not report original code. • Any additional information required to reanalyze the data reported in this paper is available from the lead contact upon request.

## Supplemental information

Document S1. Figures S1–S7 and Tables S2–S4 and S6

Table S1. Analyses of transcription factor screen by Wilcoxon-based hit calling, related to Figure 1

Table S5. Mass spectrometry data of immunoprecipitation with anti-ADNP and anti-BRG1 antibodies, related to Figure 5

Document S2. Article plus supplemental information
