# Supplementary material for: Neuroprotective protein ADNP-dependent histone remodeling complex promotes T helper 2 immune cell differentiation
Source: Immunity. 2023 Jul 11;56(7):1468–1484.e7. doi: 10.1016/j.immuni.2023.05.010 (PMC10501989; doi:10.1016/j.immuni.2023.05.010)
Supplement: Document S1. Figures S1–S7 and Tables S2–S4 and S6 [file mmc1.pdf]

Immunity, Volume *56*

Supplemental information

Neuroprotective protein ADNP-dependent histone remodeling complex promotes T helper 2 immune cell differentiation

Ana C.F. Ferreira, Aydan C.H. Szeto, Paula A. Clark, Alastair Crisp, Patrycja Kozik, Helen E. Jolin, and Andrew N.J. McKenzie

Figure S1

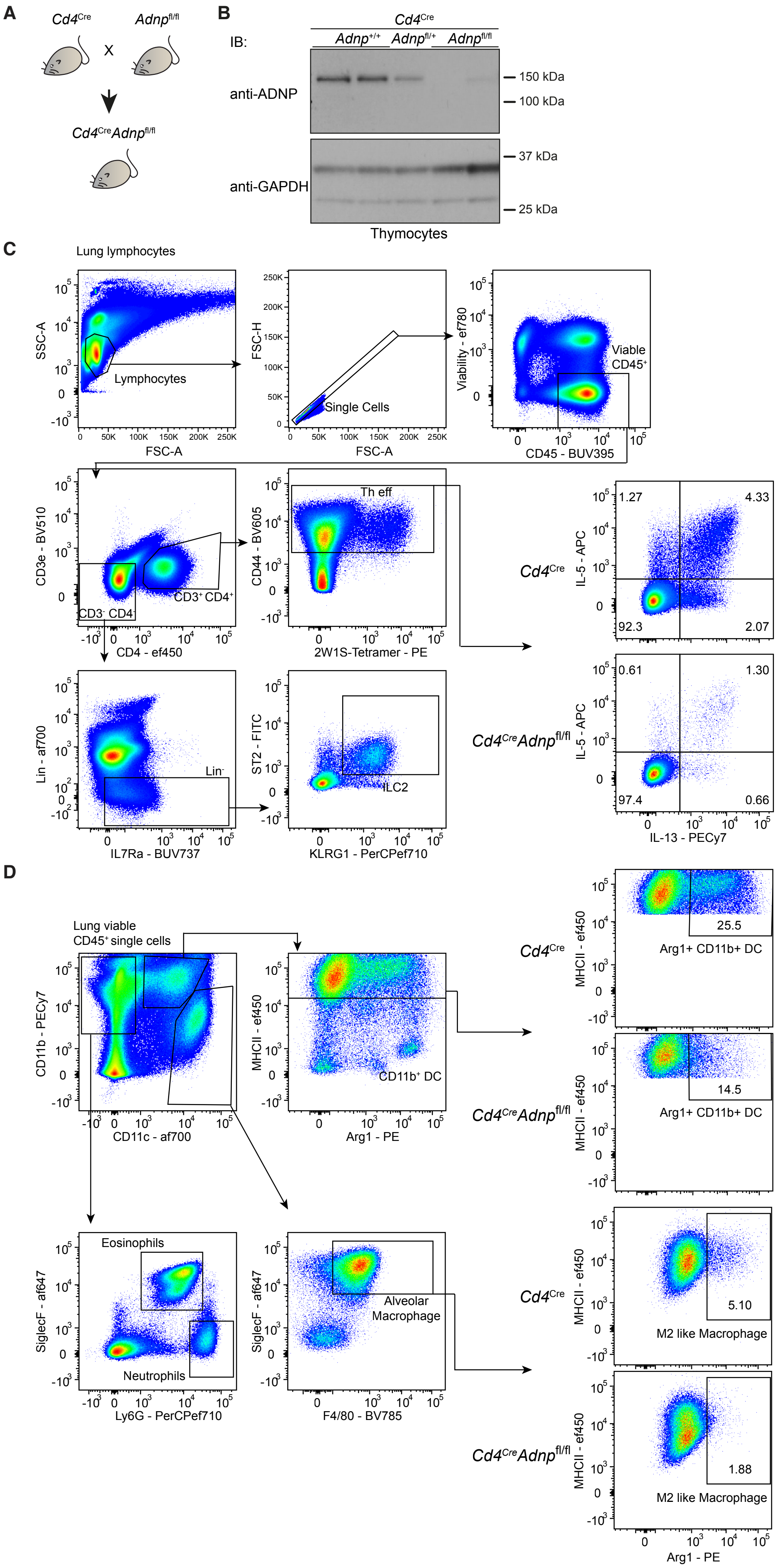
A
Cd4Cre
Adnpfl/fl
X
Cd4CreAdnpfl/fl
B
IB:
Cd4Cre
Adnp+/+
Adnpfl/+
Adnpfl/fl
anti-ADNP
150 kDa
100 kDa
37 kDa
anti-GAPDH
25 kDa
Thymocytes
C
Lung lymphocytes
SSC-A
FSC-A
Lymphocytes
FSC-H
Single Cells
Viability - ef780
Viable
CD45+
CD45 - BUV395
CD3e - BV510
CD3+ CD4+
CD3- CD4-
CD4 - ef450
CD44 - BV605
Th eff
2W1S-Tetramer - PE
Cd4Cre
IL-5 - APC
1.27
4.33
92.3
2.07
Cd4CreAdnpfl/fl
0.61
1.30
97.4
0.66
IL-13 - PECy7
Lin - af700
Lin-
IL7Ra - BUV737
ST2 - FITC
ILC2
KLRG1 - PerCPef710
D
Lung viable
CD45+ single cells
CD11b - PECy7
CD11c - af700
MHCII - ef450
CD11b+ DC
Arg1 - PE
Cd4Cre
25.5
Arg1+ CD11b+ DC
Cd4CreAdnpfl/fl
14.5
Arg1+ CD11b+ DC
SiglecF - af647
Eosinophils
Neutrophils
Ly6G - PerCPef710
Alveolar
Macrophage
F4/80 - BV785
Cd4Cre
5.10
M2 like Macrophage
Cd4CreAdnpfl/fl
1.88
M2 like Macrophage
Arg1 - PE

Figure S1. Generation of *Cd4*^{Cre}*Adnp*$^{fl/fl}$ mice and gating strategy for lymphocyte and myeloid cell population analysis, related to Figure 2.

(A) Schematic of the generation of conditional ADNP-deficient mice.

(B) Detection of ADNP (150 kDa) and GAPDH (35 kDa) proteins in purified thymocytes from the indicated mice. IB refers to immunoblotting antibody.

(C) Gating strategy for cytokine expressing lymphocytes.

(D) Gating strategy for myeloid populations.

Figure S2

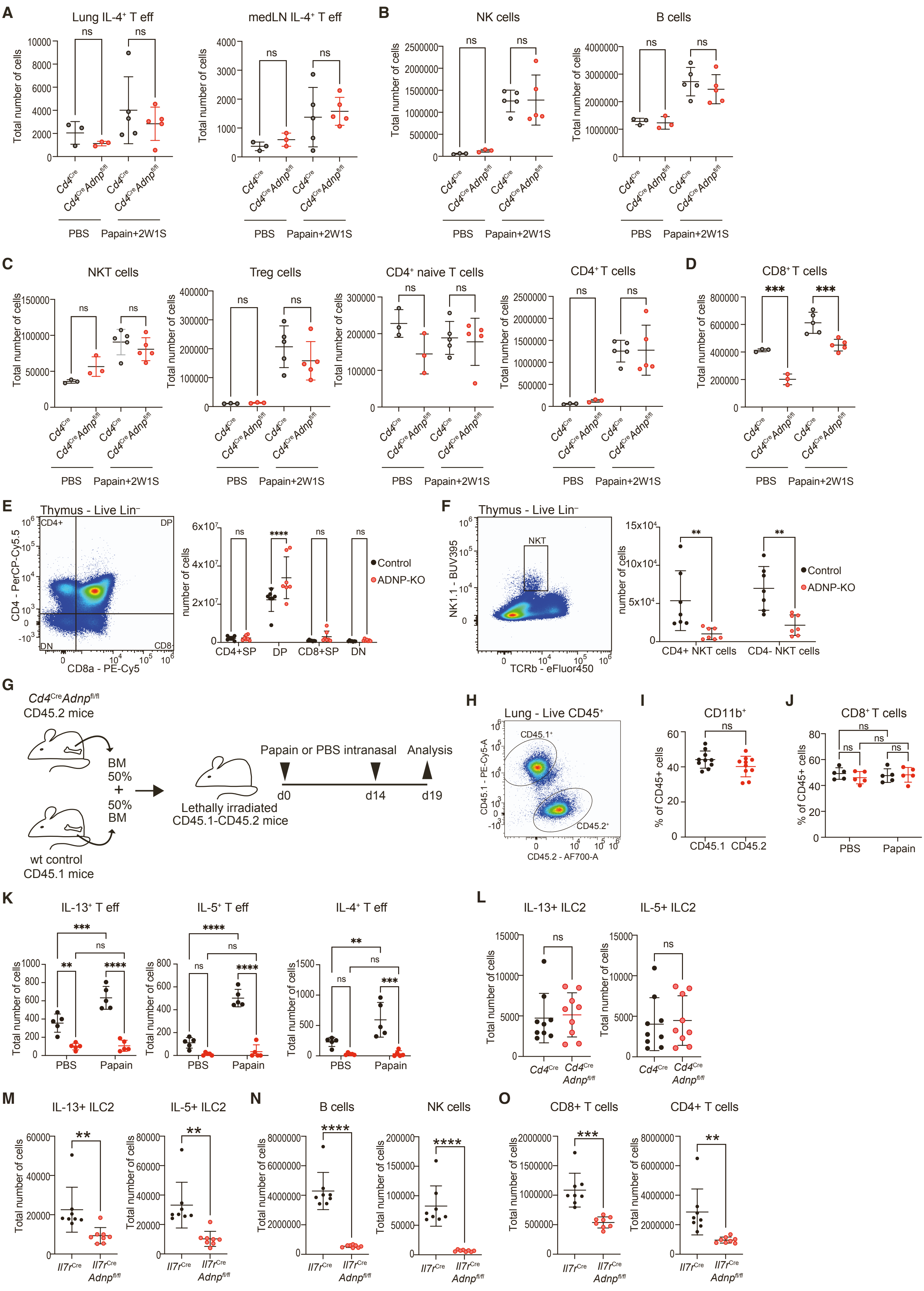

A
Lung IL-4+ T eff
medLN IL-4+ T eff
B
NK cells
B cells
Total number of cells
ns
Cd4Cre
Cd4Cre Adnpfl/fl
PBS
Papain+2W1S
C
NKT cells
Treg cells
CD4+ naive T cells
CD4+ T cells
D
CD8+ T cells
***
E
Thymus - Live Lin−
CD4+
DP
DN
CD8+
CD4 - PerCP-Cy5.5
CD8a - PE-Cy5
number of cells
****
CD4+SP
DP
CD8+SP
DN
Control
ADNP-KO
F
Thymus - Live Lin−
NKT
NK1.1 - BUV395
TCRb - eFluor450
**
CD4+ NKT cells
CD4- NKT cells
G
Cd4CreAdnpfl/fl
CD45.2 mice
BM
50%
+
50%
BM
wt control
CD45.1 mice
Lethally irradiated
CD45.1-CD45.2 mice
Papain or PBS intranasal
Analysis
d0
d14
d19
H
Lung - Live CD45+
CD45.1+
CD45.2+
CD45.1 - PE-Cy5-A
CD45.2 - AF700-A
I
CD11b+
% of CD45+ cells
CD45.1
CD45.2
J
CD8+ T cells
PBS
Papain
K
IL-13+ T eff
IL-5+ T eff
IL-4+ T eff
L
IL-13+ ILC2
IL-5+ ILC2
M
IL-13+ ILC2
IL-5+ ILC2
N
B cells
NK cells
O
CD8+ T cells
CD4+ T cells
Il7rCre
Il7rCre Adnpfl/fl

Figure S2. Lymphocyte populations following papain/2W1S challenge, related to Figure 2. (A-D) Flow cytometric analysis of (A) total lung and mediastinal lymph node IL-4-producing Th effector cells, (B) total lung NK and B cells, (C) total lung NKT, Tregs, $CD4^+$ naïve T cells and $CD4^+$ T cells, (D) total lung $CD8^+$ T cells. Comparison between *Cd4*^{Cre} mice (black dots) and *Cd4*^{Cre}*Adnp*$^{fl/fl}$ mice (red dots) treated with PBS or with papain + 2W1S peptide. Data are representative of 2 independent experiments; data are presented as mean ± SD; one-way ANOVA with Tukey's post-hoc test.

(E) Flow cytometry comparison of thymic CD4 single positive (SP), CD8 SP, CD4/CD8 double positive (DP) and double negative (DN) cells in *Cd4*^{Cre} mice (control) and *Cd4*^{Cre}*Adnp*$^{fl/fl}$ (ADNP-KO) mice. Data represent two independent experiments; mean ± SD; one-way ANOVA with Tukey's post-hoc test.

(F) Flow cytometry comparison of thymic NKT cells in *Cd4*^{Cre} mice (control) and *Cd4*^{Cre}*Adnp*$^{fl/fl}$ (ADNP-KO) mice. Data represent two independent experiments; mean ± SD; one-way ANOVA with Tukey's post-hoc test.

(G-K) Bone marrow adoptive transfer experiment.

(G) Schematic representation of the adoptive transfer of bone marrow cells from wildtype (CD45.1) and *Cd4*^{Cre}*Adnp*$^{fl/fl}$ (CD45.2) into lethally irradiated (CD45.1-CD45.2) mice followed by induction of type-2 inflammation in the mouse lung with papain.

(H) Gating strategy for $CD45.1^+$ and $CD45.2^+$ cells after adoptive transfer of bone marrow cells.

(I) Flow cytometric analysis of $CD45.1^+CD11b^+$ and $CD45.2^+CD11b^+$ cells shown as percentage of total $CD45^+$ cells. Data are presented as mean ± s.d.; paired two-sided t-test.

(J-K) Flow cytometric analysis of (J) CD8+ T cells, (K) IL-13, IL-5 and IL-4 producing Th effector cells originating from control (CD45.1 - black dots) or *Cd4*^{Cre}*Adnp*$^{fl/fl}$ (CD45.2- red dots) bone marrow cells after adoptive transfer. Data represent mean ± SD of eight mice ANOVA with Tukey's post-hoc test.

(L) Flow cytometric analysis of lung IL-13 and IL-5-producing ILC2s in *Cd4*^{Cre} (black dots) and *Cd4*^{Cre}*Adnp*$^{fl/fl}$ (red dots) mice treated with papain + 2W1S peptide. Data are representative of 2 independent experiments; data are presented as mean ± SD; one-way ANOVA with Tukey's post-hoc test.

(M) Flow cytometric analysis of lung IL-13 and IL-5-producing ILC2s in *Il7r*^{Cre} (black dots) and *Il7r*^{Cre}*Adnp*$^{fl/fl}$ (red dots) mice treated with papain + 2W1S peptide. Data are representative of 2 independent experiments; data are presented as mean ± SD; one-way ANOVA with Tukey's post-hoc test.

(N-O) Flow cytometric analysis of lung (N) B cells and NK cells, and (O) $CD8^+$ and $CD4^+$ T cells. Comparison between *Il7r*^{Cre} mice (black dots) and *Il7r*^{Cre}*Adnp*$^{fl/fl}$ mice (red dots) treated with papain + 2W1S peptide. Data are representative of 2 independent experiments; data are presented as mean ± SD; one-way ANOVA with Tukey's post-hoc test.

****P<0.0001, ***P<0.001, **P<0.01, *P<0.05, not significant (ns).

Figure S3

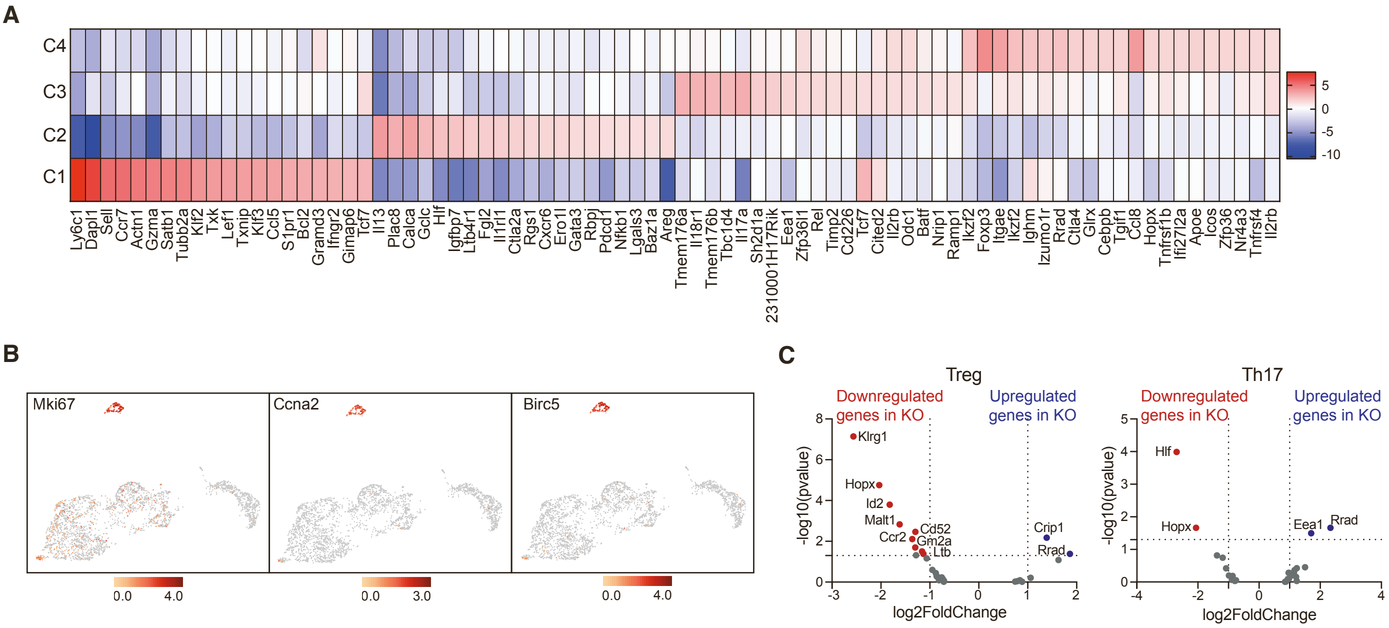
A
C4
C3
C2
C1
5
0
-5
-10
Ly6c1 Dapl1 Sell Ccr7 Actn1 Gzma Satb1 Tubb2a Klf2 Txk Lef1 Txnip Klf3 Ccl5 S1pr1 Bcl2 Gramd3 Ifngr2 Gimap6 Tcf7 Il13 Plac8 Calca Gclc Hlf Igfbp7 Ltb4r1 Fgl2 Il1rl1 Ctla2a Rgs1 Cxcr6 Ero1l Gata3 Rbpj Pdcd1 Nfkb1 Lgals3 Baz1a Areg Tmem176a Il18r1 Tmem176b Tbc1d4 Il17a Sh2d1a 2310001H17Rik Eea1 Zfp36l1 Rel Timp2 Cd226 Tcf7 Cited2 Il2rb Odc1 Batf Nrip1 Ramp1 Ikzf2 Foxp3 Itgae Ikzf2 Ighm Izumo1r Rrad Ctla4 Glrx Cebpb Tgif1 Ccl8 Hopx Tnfrsf1b Ifi27l2a Apoe Icos Zfp36 Nr4a3 Tnfrsf4 Il2rb
B
Mki67
Ccna2
Birc5
0.0 4.0
0.0 3.0
0.0 4.0
C
Treg
Downregulated genes in KO
Upregulated genes in KO
-log10(pvalue)
log2FoldChange
Klrg1
Hopx
Id2
Malt1
Cd52
Ccr2
Gm2a
Ltb
Crip1
Rrad
Th17
Downregulated genes in KO
Upregulated genes in KO
Hlf
Hopx
Eea1
Rrad

Figure S3. Single cell expression analysis of naïve and effector T cells, related to Figure 3

(A) Heatmap of the top 20 genes differentially expressed in the clusters defined in Figure 3A.

(B) UMAP plot with expression (log_2 expression) of indicated genes per individual cell.

(C) Volcano plot showing the up- and down-regulated genes comparing Th17 cells (cluster 3) and Tregs cells (cluster 4) from *Cd4*^{Cre} and *Cd4*^{Cre}*Adnp*$^{fl/fl}$ (KO) mice.

Figure S4

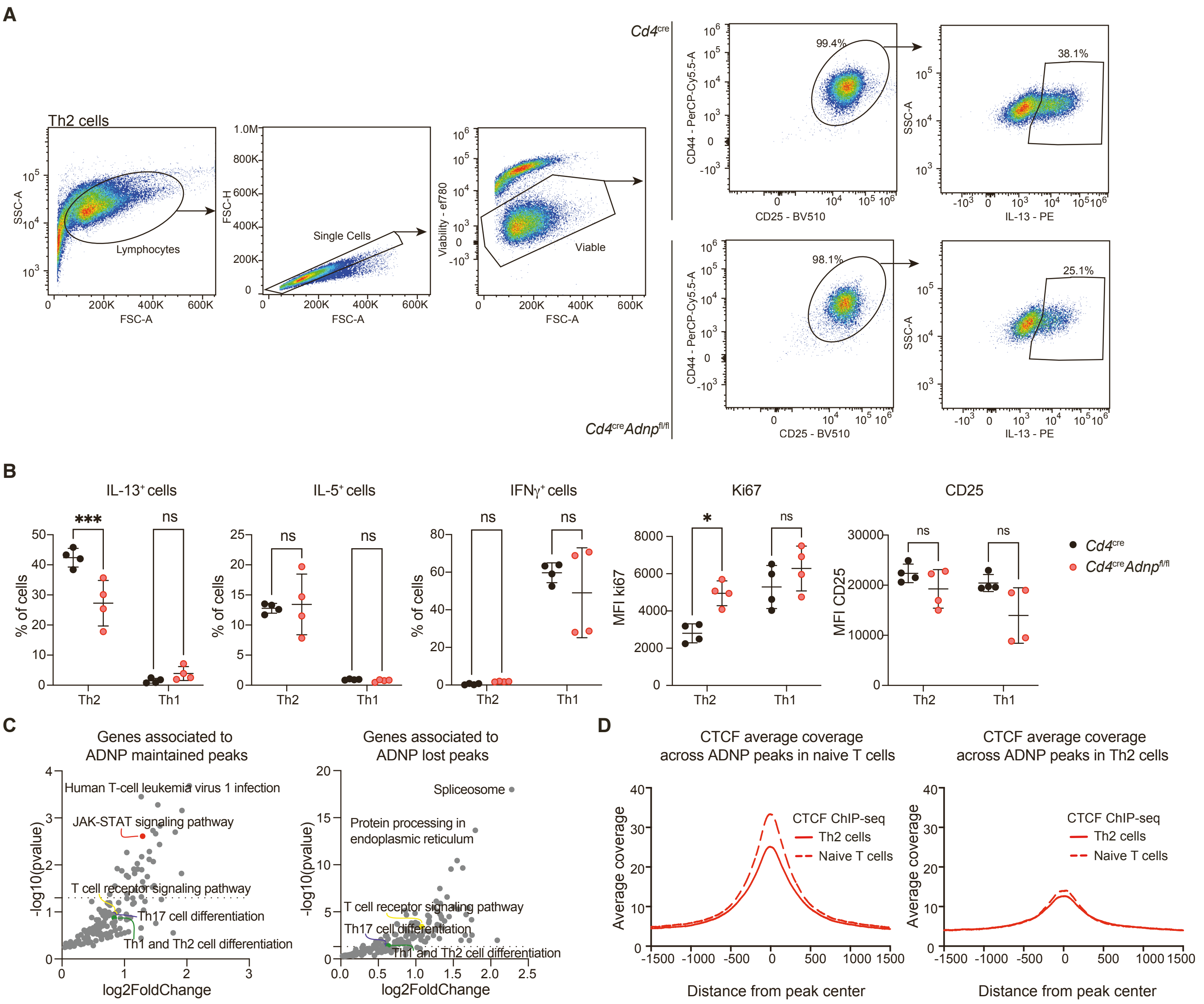

A
Th2 cells
Lymphocytes
Single Cells
Viable
SSC-A
FSC-A
FSC-H
Viability - ef780
Cd4cre
Cd4creAdnpfl/fl
99.4%
38.1%
98.1%
25.1%
CD44 - PerCP-Cy5.5-A
CD25 - BV510
IL-13 - PE
B
IL-13+ cells
IL-5+ cells
IFNγ+ cells
Ki67
CD25
% of cells
MFI ki67
MFI CD25
ns
Th2
Th1
C
Genes associated to ADNP maintained peaks
Genes associated to ADNP lost peaks
Human T-cell leukemia virus 1 infection
JAK-STAT signaling pathway
T cell receptor signaling pathway
Th17 cell differentiation
Th1 and Th2 cell differentiation
Spliceosome
Protein processing in endoplasmic reticulum
-log10(pvalue)
log2FoldChange
D
CTCF average coverage across ADNP peaks in naive T cells
CTCF average coverage across ADNP peaks in Th2 cells
CTCF ChIP-seq
Th2 cells
Naive T cells
Average coverage
Distance from peak center

Figure S4. Validation and analysis of in vitro cultured Th2 cells, related to Figure 4

(A) Gating strategy for cytokine-expressing in *in vitro* differentiated Th1 and Th2 cells.

(B) Flow cytometric analysis of Th1 and Th2 cells after *in vitro* differentiation. Data are shown as percentage of $CD44^{+}CD25^{+}$ and median fluorescence intensity (MFI) and are presented as mean ± s.d.; one-way ANOVA with Tukey's post-hoc test. ****P<0.0001, *P<0.05, not significant (ns).

(C) KEGG pathway analysis of the genes associated with ADNP maintained peaks (peaks present in Th2 cells and in naïve T cells); and KEGG pathway analysis of the genes associated with ADNP lost peaks (peaks present in naïve T cells but not in Th2 cells). All pathways shown were enriched (p<0.05).

(D) Average CTCF ChIP-seq signal across ADNP peaks in naïve T cells and across ADNP peaks in Th2 cells. Data representative of 2 biological replicates.

Figure S5

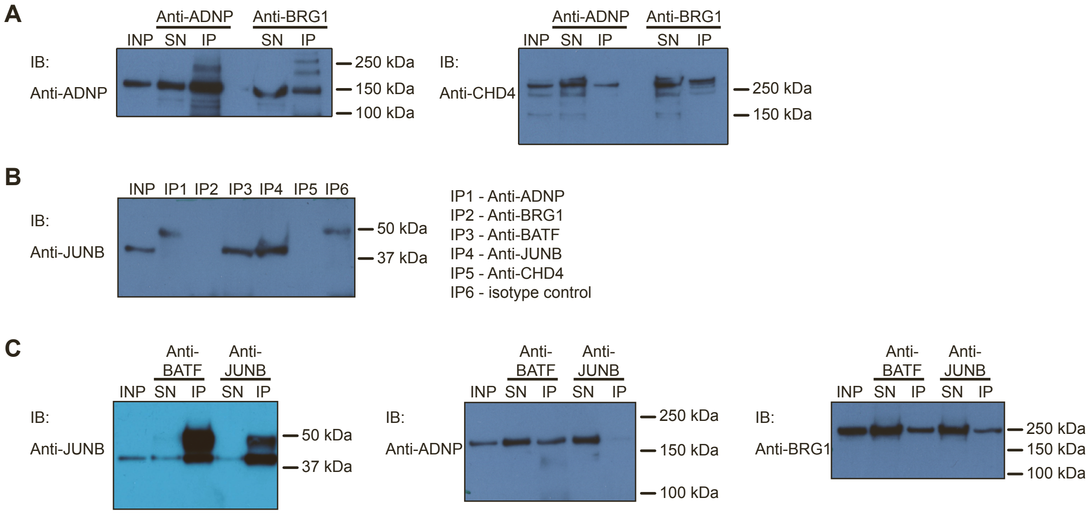
A
Anti-ADNP
Anti-BRG1
INP SN IP SN IP
IB:
Anti-ADNP
250 kDa
150 kDa
100 kDa
Anti-ADNP
Anti-BRG1
INP SN IP SN IP
IB:
Anti-CHD4
250 kDa
150 kDa
B
INP IP1 IP2 IP3 IP4 IP5 IP6
IB:
Anti-JUNB
50 kDa
37 kDa
IP1 - Anti-ADNP
IP2 - Anti-BRG1
IP3 - Anti-BATF
IP4 - Anti-JUNB
IP5 - Anti-CHD4
IP6 - isotype control
C
Anti-BATF
Anti-JUNB
INP SN IP SN IP
IB:
Anti-JUNB
50 kDa
37 kDa
Anti-BATF
Anti-JUNB
INP SN IP SN IP
IB:
Anti-ADNP
250 kDa
150 kDa
100 kDa
Anti-BATF
Anti-JUNB
INP SN IP SN IP
IB:
Anti-BRG1
250 kDa
150 kDa
100 kDa

Figure S5. ADNP forms complexes with BRG1 and BATF in Th2 cells, related to Figure 5.

(A-C) Lanes were loaded with nuclear extract before immunoprecipitation (INP), supernatant of the immunoprecipitation (SN) or elution of the immunoprecipitation (IP). IB refers to immunoblotting antibody. Data are representative of 2 independent experiments.

(A) Protein associations confirmed by co-immunoprecipitation experiments. Detection of ADNP (150 kDa) and CHD4 (226 kDa) proteins in immunocomplexes generated with Th2 cell nuclear lysate co-immunoprecipitated with anti-ADNP or anti-BRG1 antibodies.

(B) Confirmed of protein associations using co-immunoprecipitation. Detection of JUNB (38 kDa) protein in immunocomplexes generated with Th2 cell nuclear lysate co-immunoprecipitated with anti-ADNP, anti-BRG1, anti-BATF, anti-JUNB, anti-CHD4 or isotype control antibodies.

(C) Confirmed of protein associations using co-immunoprecipitation. Detection of JUNB (38 kDa), ADNP (150 kDa) and BRG1 (181 kDa) proteins in immunocomplexes generated with Th2 cell nuclear lysate co-immunoprecipitated with anti-BATF or anti-JUNB antibodies.

Figure S6

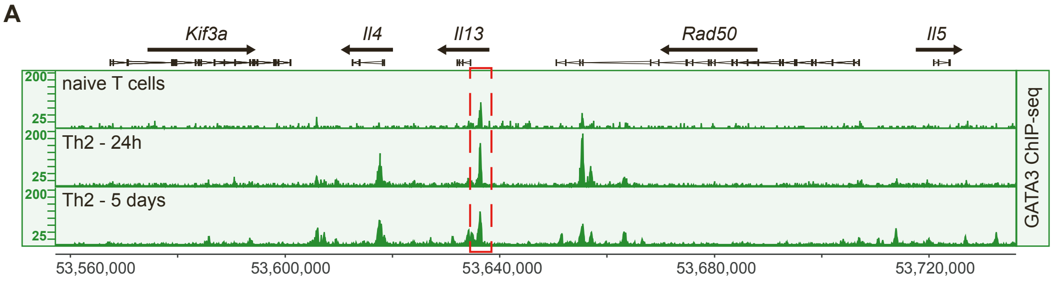
A
Kif3a
Il4
Il13
Rad50
Il5
200
25
naive T cells
Th2 - 24h
Th2 - 5 days
GATA3 ChIP-seq
53,560,000
53,600,000
53,640,000
53,680,000
53,720,000

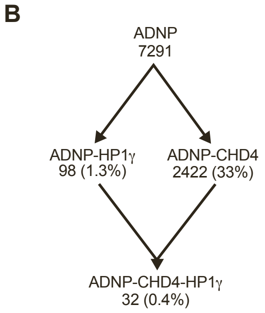
B
ADNP
7291
ADNP-HP1γ
98 (1.3%)
ADNP-CHD4
2422 (33%)
ADNP-CHD4-HP1γ
32 (0.4%)

Figure S6. GATA3 binds to the CGRE before ADNP recruitment, related to Figure 6

(A) Representative binding profiles of GATA3 in wild type naïve T cells, *Cd4*^{Cre} Th2 cells and *Cd4*^{Cre}*Adnp*$^{fl/fl}$ Th2 cells in the type-2 cytokine locus. Data representative of 2 biological replicates.

(B) Number and percentage of all ADNP, ADNP-HP1γ, ADNP-CHD4 and ADNP-CHD4- HP1γ overlapping ChIP-seq peaks in Th2 cells. Peak list was generated using two biological replicates.

Figure S7

A

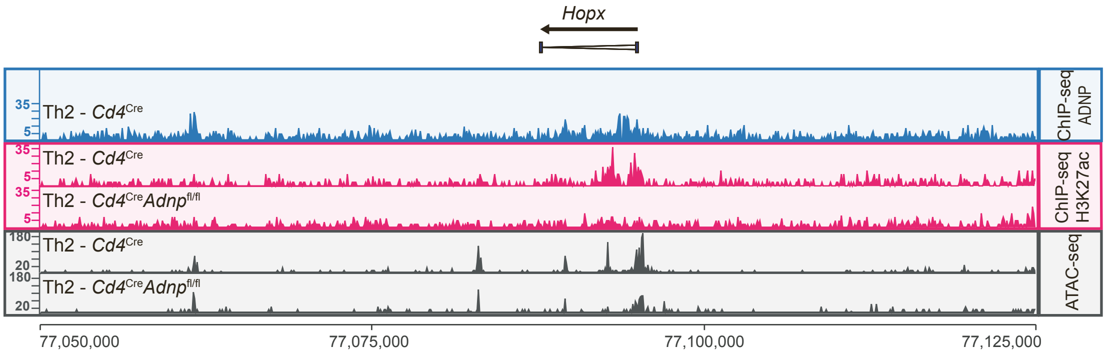

Hopx
35
5
Th2 - Cd4Cre
ChIP-seq ADNP
Th2 - Cd4Cre
Th2 - Cd4CreAdnpfl/fl
ChIP-seq H3K27ac
180
20
Th2 - Cd4Cre
Th2 - Cd4CreAdnpfl/fl
ATAC-seq
77,050,000
77,075,000
77,100,000
77,125,000

Figure S7. ChIP-seq and ATAC-seq analysis of *Hopx* locus, related to Figure 7

(A) Representative ADNP and H3K27ac ChIP-seq, and ATAC-seq tracks in Th2 cells from *Cd4*^{Cre} or *Cd4*^{cre}*Adnp*$^{fl/fl}$ mice at the *Hopx* locus. Data representative of 2 biological replicates.

Table S2. Differentially expressed genes in RNA-seq data comparing Th2 cells following Adnp-deletion with non-deleted cells, related to Figure 1.

Upregulated genes			
Gene symbol	Gene name	P-value	Fold change
ENSMUSG00000024592	*C330018D20Rik*	0.00	-27.15
ENSMUSG00000085823	*Gm13012*	0.00	-18.44
ENSMUSG00000002731	*Prkra*	0.00	-18.15
ENSMUSG00000003271	*Sult2b1*	0.01	-9.97
ENSMUSG00000025008	*Tctn3*	0.01	-9.43
ENSMUSG00000029575	*Mmab*	0.02	-9.03
ENSMUSG00000025921	*Rdh10*	0.01	-8.91
ENSMUSG00000042178	*Armc5*	0.00	-8.70
ENSMUSG00000034105	*Meak7*	0.03	-7.90
ENSMUSG00000034875	*Nudt19*	0.01	-7.68
ENSMUSG00000042363	*Lgalsl*	0.01	-7.48
ENSMUSG00000062190	*Lancl2*	0.00	-7.37
ENSMUSG00000049410	*Zfp683*	0.01	-6.83
ENSMUSG00000091649	*Phf11b*	0.01	-6.77
ENSMUSG00000026433	*Rab29*	0.00	-6.73
ENSMUSG00000098322	*5830468F06Rik*	0.02	-6.73
ENSMUSG00000043079	*Synpo*	0.02	-6.25
ENSMUSG00000029196	*Tada2b*	0.03	-6.15
ENSMUSG00000074738	*Fndc10*	0.02	-5.98
ENSMUSG00000040498	*Igsf23*	0.03	-5.89
ENSMUSG00000116858	*Gm49797*	0.05	-5.72
ENSMUSG00000029084	*Cd38*	0.05	-5.51
ENSMUSG00000015189	*Casd1*	0.01	-5.19
ENSMUSG00000018919	*Tm4sf5*	0.03	-5.06
ENSMUSG00000095123	*Gm21781*	0.00	-5.05
ENSMUSG00000073771	*Btbd19*	0.00	-5.04
ENSMUSG00000001552	*Jup*	0.05	-4.88
ENSMUSG00000037405	*Icam1*	0.01	-4.84
ENSMUSG00000030443	*Zfp583*	0.03	-4.60
ENSMUSG00000002825	*Qtrt1*	0.04	-4.36
ENSMUSG00000038352	*Arl5c*	0.01	-4.35
ENSMUSG00000054752	*Fsd1l*	0.02	-4.20
ENSMUSG00000052676	*Zmat1*	0.04	-4.13
ENSMUSG00000040557	*Mettl27*	0.01	-4.10
ENSMUSG00000039108	*Lsm14b*	0.02	-4.00
ENSMUSG00000040652	*Oaz2*	0.02	-3.98
ENSMUSG00000106005	*Gm29151*	0.04	-3.87
ENSMUSG00000042262	*Ccr8*	0.02	-3.78
ENSMUSG00000045438	*Cox19*	0.04	-3.70
ENSMUSG00000117219	*Gm49911*	0.05	-3.70
ENSMUSG00000030612	*Mrpl46*	0.02	-3.67
ENSMUSG00000035840	*Lysmd3*	0.02	-3.66
ENSMUSG00000044881	*Coa4*	0.05	-3.64
ENSMUSG00000032067	*Pts*	0.04	-3.63
ENSMUSG00000021271	*Zfp839*	0.00	-3.61
ENSMUSG00000028690	*Mmachc*	0.02	-3.60
ENSMUSG00000034858	*Fam214a*	0.04	-3.53
ENSMUSG00000053477	*Tcf4*	0.03	-3.52
ENSMUSG00000017631	*Abr*	0.01	-3.43
ENSMUSG00000016756	*Cmah*	0.00	-3.34
ENSMUSG00000040929	*Rfx3*	0.01	-3.34
ENSMUSG00000109652	*Gm45555*	0.04	-3.30
ENSMUSG00000002205	*Vrk3*	0.03	-3.25
ENSMUSG00000087141	*Plcxd2*	0.04	-3.24
ENSMUSG00000025997	*Ikzf2*	0.01	-3.22
ENSMUSG00000036327	*Qsox2*	0.00	-3.16
ENSMUSG00000005897	*Nr2c1*	0.03	-3.13
ENSMUSG00000025979	*Mob4*	0.01	-3.10
ENSMUSG00000073096	*Lrrc61*	0.00	-3.10
ENSMUSG00000037824	*Tspan14*	0.02	-3.10
ENSMUSG00000026810	*Dpm2*	0.02	-3.08
ENSMUSG00000041143	*Tmco4*	0.02	-3.06
ENSMUSG00000038034	*Igsf8*	0.02	-3.05
ENSMUSG00000026018	*Ica1l*	0.05	-3.02
ENSMUSG00000033706	*Smyd5*	0.02	-2.97
ENSMUSG00000024588	*Fech*	0.01	-2.97
ENSMUSG00000024778	*Fas*	0.02	-2.92
ENSMUSG00000050079	*Rspry1*	0.01	-2.88
ENSMUSG00000025078	*Nhlrc2*	0.02	-2.88
ENSMUSG00000028990	*Lzic*	0.03	-2.84
ENSMUSG00000028655	*Mfsd2a*	0.01	-2.83
ENSMUSG00000038387	*Rras*	0.01	-2.72
ENSMUSG00000027495	*Fam210b*	0.03	-2.68
ENSMUSG00000060733	*Ipmk*	0.02	-2.64
ENSMUSG00000059149	*Mfsd4a*	0.04	-2.62
ENSMUSG00000067629	*Syngap1*	0.02	-2.55

Downregulated genes			
Gene symbol	Gene name	P-value	Fold change
ENSMUSG00000001700	*Gramd3*	0.00	44.63
ENSMUSG00000104795	*Gm42783*	0.00	32.95
ENSMUSG00000020099	*Unc5b*	0.00	20.78
ENSMUSG00000018648	*Dusp14*	0.00	20.75
ENSMUSG00000033703	*Fcsk*	0.02	19.27
ENSMUSG00000074064	*Mlycd*	0.00	16.08
ENSMUSG00000030200	*Bcl2l14*	0.02	15.25
ENSMUSG00000109864	*Eid3*	0.03	13.81
ENSMUSG00000036882	*Arhgap33*	0.00	13.70
ENSMUSG00000021203	*Otub2*	0.01	12.69
ENSMUSG00000020122	*Egfr*	0.00	12.65
ENSMUSG00000109379	*Gm44550*	0.01	12.61
ENSMUSG00000030406	*Gipr*	0.00	12.40
ENSMUSG00000021485	*Mxd3*	0.01	12.33
ENSMUSG00000104698	*Gm42602*	0.02	11.91
ENSMUSG00000003812	*Dnase2a*	0.00	11.81
ENSMUSG00000097727	*F630040K05Rik*	0.01	11.49
ENSMUSG00000040272	*Accs*	0.05	11.21
ENSMUSG00000112096	*A430103D13Rik*	0.01	11.06
ENSMUSG00000037716	*Ccdc33*	0.00	10.49
ENSMUSG00000032109	*Nlrx1*	0.01	10.19
ENSMUSG00000028238	*Atp6v0d2*	0.02	10.17
ENSMUSG00000020261	*Slc36a1*	0.04	9.77
ENSMUSG00000106241	*Gm43143*	0.02	9.24
ENSMUSG00000030688	*Stard10*	0.00	8.96
ENSMUSG00000044934	*Zfp367*	0.00	8.35
ENSMUSG00000104302	*Gm36975*	0.01	8.20
ENSMUSG00000058258	*Idi1*	0.01	7.91
ENSMUSG00000034903	*Cobll1*	0.00	7.54
ENSMUSG00000028217	*Cdh17*	0.01	7.41
ENSMUSG00000020021	*Fgd6*	0.03	7.39
ENSMUSG00000031969	*Acad8*	0.02	7.20
ENSMUSG00000033147	*Slc22a15*	0.01	7.11
ENSMUSG00000028582	*Cc2d1b*	0.00	7.05
ENSMUSG00000005043	*Sgsh*	0.03	6.81
ENSMUSG00000030613	*Ccdc90b*	0.01	6.77
ENSMUSG00000053007	*Creb5*	0.01	6.72
ENSMUSG00000079043	*Fastkd5*	0.02	6.58
ENSMUSG00000001065	*Zfp276*	0.00	6.47
ENSMUSG00000035439	*Haus8*	0.03	6.44
ENSMUSG00000013833	*Med16*	0.02	6.43
ENSMUSG00000104239	*Gm37588*	0.05	6.41
ENSMUSG00000062729	*Ppox*	0.01	6.30
ENSMUSG00000087574	*C030037D09Rik*	0.02	6.07
ENSMUSG00000041298	*Katnal1*	0.02	6.07
ENSMUSG00000050323	*Ndufaf6*	0.02	5.94
ENSMUSG00000104342	*Gm36401*	0.02	5.93
ENSMUSG00000043895	*S1pr2*	0.00	5.87
ENSMUSG00000041540	*Sox5*	0.00	5.80
ENSMUSG00000097300	*Sell*	0.01	5.45
ENSMUSG00000037628	*Cdkn3*	0.03	5.23
ENSMUSG00000071547	*Nt5dc2*	0.03	5.22
ENSMUSG00000025492	*Ifitm3*	0.04	5.16
ENSMUSG00000032754	*Slc8b1*	0.03	5.11
ENSMUSG00000003518	*Dusp3*	0.02	4.89
ENSMUSG00000039450	*Dcxr*	0.01	4.70
ENSMUSG00000029821	*Gsdme*	0.00	4.69
ENSMUSG00000038301	*Snx10*	0.00	4.61
ENSMUSG00000048120	*Entpd1*	0.04	4.61
ENSMUSG00000035640	*Cbarp*	0.03	4.57
ENSMUSG00000024070	*Prkd3*	0.02	4.49
ENSMUSG00000037949	*Ano10*	0.03	4.49
ENSMUSG00000091586	*Cyp4f17*	0.01	4.47
ENSMUSG00000054770	*Kctd18*	0.02	4.47
ENSMUSG00000018916	*Csf2*	0.03	4.46
ENSMUSG00000011254	*Thg1l*	0.01	4.40
ENSMUSG00000021285	*Ppp1r13b*	0.03	4.31
ENSMUSG00000055866	*Per2*	0.03	4.31
ENSMUSG00000022160	*Mettl3*	0.04	4.24
ENSMUSG00000042705	*Commd10*	0.01	4.24
ENSMUSG00000058886	*Deaf1*	0.04	4.19
ENSMUSG00000006191	*Cdkal1*	0.00	4.17
ENSMUSG00000030880	*Polr3e*	0.03	4.17
ENSMUSG00000040596	*Pogk*	0.01	4.16
ENSMUSG00000104339	*C130089K02Rik*	0.02	4.14
ENSMUSG00000026698	*Pigc*	0.02	4.06

ENSMUSG00000062202	*Btbd9*	0.05	-2.54
ENSMUSG00000073684	*Faap20*	0.04	-2.52
ENSMUSG00000038119	*Cdon*	0.00	-2.52
ENSMUSG00000075376	*Rc3h2*	0.01	-2.51
ENSMUSG00000028394	*Pole3*	0.04	-2.50
ENSMUSG00000039298	*Cdk5rap2*	0.04	-2.45
ENSMUSG00000074604	*Mgst2*	0.02	-2.43
ENSMUSG00000103175	*Gm37169*	0.00	-2.43
ENSMUSG00000073008	*Gpr174*	0.00	-2.43
ENSMUSG00000028341	*Nr4a3*	0.02	-2.42
ENSMUSG00000030965	*Abraxas2*	0.03	-2.39
ENSMUSG00000026483	*Fam129a*	0.02	-2.38
ENSMUSG00000011382	*Dhdh*	0.01	-2.37
ENSMUSG00000024370	*Cdc23*	0.00	-2.37
ENSMUSG00000056708	*Ier5*	0.03	-2.34
ENSMUSG00000003731	*Kpna6*	0.03	-2.33
ENSMUSG00000001755	*Coasy*	0.04	-2.31
ENSMUSG00000071647	*Eml3*	0.04	-2.27
ENSMUSG00000042111	*Ccdc115*	0.00	-2.27
ENSMUSG00000038002	*Cramp1l*	0.01	-2.27
ENSMUSG00000030493	*Faap24*	0.04	-2.27
ENSMUSG00000020346	*Mgat1*	0.05	-2.26
ENSMUSG00000023965	*Fbxl17*	0.01	-2.26
ENSMUSG00000040123	*Zmym5*	0.01	-2.22
ENSMUSG00000041815	*Poldip3*	0.03	-2.21
ENSMUSG00000031711	*Zfp330*	0.05	-2.19
ENSMUSG00000074480	*Mex3a*	0.03	-2.19
ENSMUSG00000028599	*Tnfrsf1b*	0.03	-2.18
ENSMUSG00000064289	*Tank*	0.00	-2.18
ENSMUSG00000035772	*Mrps2*	0.05	-2.18
ENSMUSG00000023460	*Rab12*	0.04	-2.17
ENSMUSG00000049232	*Tigd2*	0.04	-2.15
ENSMUSG00000030264	*Thumpd3*	0.03	-2.15
ENSMUSG00000042312	*S100a13*	0.01	-2.13
ENSMUSG00000043510	*Hscb*	0.03	-2.12
ENSMUSG00000027351	*Spred1*	0.04	-2.12
ENSMUSG00000053134	*Supt7l*	0.00	-2.06
ENSMUSG00000041225	*Arhgap12*	0.01	-2.05
ENSMUSG00000070923	*Klhl9*	0.02	-2.04
ENSMUSG00000032115	*Hyou1*	0.02	-2.03
ENSMUSG00000032612	*Usp4*	0.04	-2.02

ENSMUSG00000028199	*Cryz*	0.01	4.05
ENSMUSG00000018914	*Il3*	0.02	4.00
ENSMUSG00000034973	*Dop1a*	0.00	3.95
ENSMUSG00000027459	*Fam110a*	0.01	3.93
ENSMUSG00000015944	*Castor2*	0.00	3.92
ENSMUSG00000021461	*Fancc*	0.03	3.87
ENSMUSG00000027864	*Ptgfrn*	0.00	3.87
ENSMUSG00000047242	*Taf9b*	0.03	3.85
ENSMUSG00000076761	*Trav5-1*	0.02	3.85
ENSMUSG00000007603	*Dus3l*	0.04	3.83
ENSMUSG00000059866	*Tnip2*	0.01	3.81
ENSMUSG00000040888	*Gfer*	0.01	3.74
ENSMUSG00000046731	*Kctd11*	0.01	3.73
ENSMUSG00000033902	*Mapkbp1*	0.03	3.72
ENSMUSG00000087384	*Gm15558*	0.03	3.68
ENSMUSG00000033632	*AW554918*	0.04	3.67
ENSMUSG00000028073	*Pear1*	0.05	3.67
ENSMUSG00000020741	*Cluh*	0.03	3.67
ENSMUSG00000030600	*Lrfn1*	0.03	3.63
ENSMUSG00000110206	*Flt3l*	0.02	3.57
ENSMUSG00000039474	*Wfs1*	0.04	3.53
ENSMUSG00000021703	*Serinc5*	0.00	3.53
ENSMUSG00000015647	*Lama5*	0.00	3.52
ENSMUSG00000037816	*Fbxw17*	0.03	3.51
ENSMUSG00000031802	*Phxr4*	0.02	3.49
ENSMUSG00000022479	*Vdr*	0.01	3.49
ENSMUSG00000000441	*Raf1*	0.01	3.48
ENSMUSG00000037239	*Spred3*	0.01	3.46
ENSMUSG00000030203	*Dusp16*	0.00	3.39
ENSMUSG00000038542	*Pcid2*	0.00	3.36
ENSMUSG00000074151	*Nlrc5*	0.01	3.35
ENSMUSG00000066149	*Cdc26*	0.02	3.35
ENSMUSG00000026274	*Pask*	0.02	3.34
ENSMUSG00000026014	*Raph1*	0.00	3.31
ENSMUSG00000047751	*Utf1*	0.04	3.25
ENSMUSG00000032041	*Tirap*	0.00	3.22
ENSMUSG00000023235	*Cd25*	0.02	3.19
ENSMUSG00000020537	*Drg2*	0.02	3.18
ENSMUSG00000022246	*Rai14*	0.03	3.17
ENSMUSG00000025743	*Sdc3*	0.01	3.15
ENSMUSG00000006058	*Snf8*	0.00	3.13
ENSMUSG00000055435	*Maf*	0.01	3.13
ENSMUSG00000027293	*Ehd4*	0.00	3.12
ENSMUSG00000033880	*Lgals3bp*	0.03	3.07
ENSMUSG00000109005	*Gm45221*	0.02	3.05
ENSMUSG00000033323	*Ctdp1*	0.02	3.04
ENSMUSG00000020453	*Patz1*	0.05	3.04
ENSMUSG00000000869	*Il4*	0.00	3.01
ENSMUSG00000031913	*Vps4a*	0.01	3.01
ENSMUSG00000022668	*Gtpbp8*	0.05	3.01
ENSMUSG00000054676	*1600014C10Rik*	0.02	3.00
ENSMUSG00000033629	*Hacd3*	0.03	2.99
ENSMUSG00000102336	*Gm37233*	0.00	2.98
ENSMUSG00000096188	*Cmtm4*	0.03	2.92
ENSMUSG00000026766	*Mmadhc*	0.01	2.90
ENSMUSG00000031577	*Tti2*	0.03	2.88
ENSMUSG00000028683	*Eif2b3*	0.03	2.85
ENSMUSG00000029387	*Gtf2h3*	0.01	2.85
ENSMUSG00000039959	*Hip1*	0.01	2.83
ENSMUSG00000024781	*Lipa*	0.01	2.82
ENSMUSG00000059895	*Ptp4a3*	0.01	2.81
ENSMUSG00000043671	*Dpy19l3*	0.05	2.78
ENSMUSG00000079215	*Zfp664*	0.02	2.75
ENSMUSG00000023951	*Vegfa*	0.00	2.75
ENSMUSG00000031266	*Gla*	0.02	2.68
ENSMUSG00000029176	*Anapc4*	0.00	2.68
ENSMUSG00000035790	*Cep19*	0.01	2.66
ENSMUSG00000023286	*Ube2j2*	0.02	2.66
ENSMUSG00000024829	*Mrpl21*	0.01	2.65
ENSMUSG00000016028	*Celsr1*	0.00	2.64
ENSMUSG00000032601	*Prkar2a*	0.00	2.63
ENSMUSG00000034187	*Nsf*	0.00	2.63
ENSMUSG00000021629	*Slc30a5*	0.04	2.60
ENSMUSG00000002797	*Ggct*	0.05	2.55
ENSMUSG00000061981	*Flot2*	0.02	2.54
ENSMUSG00000028559	*Osbpl9*	0.04	2.54
ENSMUSG00000027447	*Cst3*	0.03	2.54
ENSMUSG00000026749	*Nek6*	0.00	2.47
ENSMUSG00000056498	*Tmem154*	0.02	2.46
ENSMUSG00000026830	*Ermn*	0.01	2.44
ENSMUSG00000042625	*Safb2*	0.04	2.44

ENSMUSG00000020948	*Klhl28*	0.03	2.42
ENSMUSG00000103391	*Gm38302*	0.04	2.41
ENSMUSG00000030469	*Zfp719*	0.04	2.41
ENSMUSG00000029401	*Rilpl2*	0.00	2.39
ENSMUSG00000047454	*Gphn*	0.01	2.37
ENSMUSG00000040462	*Os9*	0.02	2.36
ENSMUSG00000037373	*Ctbp1*	0.04	2.36
ENSMUSG00000039153	*Runx2*	0.01	2.32
ENSMUSG00000015536	*Mocs2*	0.03	2.31
ENSMUSG00000030990	*Pgap2*	0.04	2.30
ENSMUSG00000042726	*Trafd1*	0.01	2.27
ENSMUSG00000026568	*Mpc2*	0.03	2.26
ENSMUSG00000104291	*A130071D04Rik*	0.04	2.26
ENSMUSG00000043154	*Ppp2r3a*	0.01	2.26
ENSMUSG00000062270	*Morf4l1*	0.01	2.25
ENSMUSG00000064181	*Rab3ip*	0.02	2.25
ENSMUSG00000000194	*Gpr107*	0.01	2.25
ENSMUSG00000018398	*Septin8*	0.03	2.22
ENSMUSG00000024130	*Abca3*	0.03	2.21
ENSMUSG00000030659	*Nucb2*	0.01	2.20
ENSMUSG00000039318	*Rab3gap2*	0.01	2.18
ENSMUSG00000034021	*Pds5b*	0.02	2.17
ENSMUSG00000038214	*Bend3*	0.02	2.15
ENSMUSG00000024007	*Ppil1*	0.05	2.15
ENSMUSG00000029910	*Mad2l1*	0.02	2.14
ENSMUSG00000013662	*Atad1*	0.04	2.12
ENSMUSG00000022685	*Parn*	0.01	2.11
ENSMUSG00000028772	*Zcchc17*	0.03	2.09
ENSMUSG00000057649	*Brd9*	0.03	2.07
ENSMUSG00000031813	*Mvb12a*	0.04	2.05
ENSMUSG00000030286	*Emc3*	0.00	2.02
ENSMUSG00000011179	*Odc1*	0.03	2.02
ENSMUSG00000019979	*Apaf1*	0.01	2.00

Table S3. KEGG pathway analysis of the genes associated to ADNP peaks in Th2 cells, related to Figure 4.

Term	log2FoldChange	-Log10(pvalue)
Pathways in cancer	0.896310577179861	8.899639752
Th1 and Th2 cell differentiation	1.98043531241309	8.753305558
Th17 cell differentiation	1.76564716172519	8.013879289
JAK-STAT signaling pathway	1.4130646456827	7.815124537
T cell receptor signaling pathway	1.67047451480334	7.106680533
Thyroid hormone signaling pathway	1.4815761505588	6.279771765
p53 signaling pathway	1.74194628189252	5.655094698
Hepatitis B	1.18603154380679	5.508270982
Human T-cell leukemia virus 1 infection	0.930648014501768	4.916323398
Osteoclast differentiation	1.24399800998024	4.910145577
Small cell lung cancer	1.43824161586732	4.886122366
MAPK signaling pathway	0.83799240009741	4.713395787
Regulation of actin cytoskeleton	0.958481354228799	4.693042021
Chronic myeloid leukemia	1.49772800702011	4.484146972
Measles	1.0931439309215	4.255398949
Apoptosis	1.03976730156872	3.816990684
Adherens junction	1.36342198494941	3.598004337
Proteoglycans in cancer	0.849943835879489	3.578042046
Leukocyte transendothelial migration	1.08285576196112	3.478885214
Human cytomegalovirus infection	0.731122775503737	3.301206219
Inflammatory bowel disease (IBD)	1.41764126080508	3.279119638
Colorectal cancer	1.17475112426983	3.240442986
Rap1 signaling pathway	0.792083167191249	3.239806306
NF-kappa B signaling pathway	1.07904334898118	3.13968771
C-type lectin receptor signaling pathway	1.01535434729982	3.041471096
Neurotrophin signaling pathway	0.977993785339682	3.029805967
Hepatocellular carcinoma	0.834003571851602	3.024282206
Non-small cell lung cancer	1.2731495194613	2.964906282
Pancreatic cancer	1.18591979537282	2.8994984
FoxO signaling pathway	0.914632842384355	2.891957942
Chemokine signaling pathway	0.759109753219616	2.879640638
Kaposi sarcoma-associated herpesvirus infection	0.727337176682901	2.877866889
Toxoplasmosis	0.961478897746632	2.693409514
cAMP signaling pathway	0.700445526377739	2.657193068
Insulin resistance	0.92430963778094	2.55753113
TNF signaling pathway	0.92430963778094	2.55753113
PI3K-Akt signaling pathway	0.532652061495029	2.532664588
Epstein-Barr virus infection	0.648991410107192	2.497879794
Leishmaniasis	1.14130813421849	2.485799551
Renal cell carcinoma	1.11019387930504	2.400657166
Wnt signaling pathway	0.737362282365623	2.347898844
Viral carcinogenesis	0.614166505725594	2.290136317
Insulin signaling pathway	0.757334445863519	2.208541569
Platelet activation	0.794434780249947	2.203015932
Glioma	1.00387435465186	2.192117
Oxytocin signaling pathway	0.714042250439738	2.170618437
Focal adhesion	0.621350310771721	2.10755219
B cell receptor signaling pathway	0.992007898931744	2.086443427
Natural killer cell mediated cytotoxicity	0.78444646769435	2.071043345
Breast cancer	0.701813889111462	2.043961956
AGE-RAGE signaling pathway in diabetic complications	0.81807349240271	1.975130928
Transcriptional misregulation in cancer	0.614636906867805	1.954106558
Hippo signaling pathway	0.652203023301662	1.934391062
Vascular smooth muscle contraction	0.688348256117813	1.91740868
Aldosterone synthesis and secretion	0.798620659875491	1.91724637
Parathyroid hormone synthesis, secretion and action	0.776198830064104	1.899781426
Hepatitis C	0.640142629131631	1.889724583
Cellular senescence	0.593850913771571	1.871385917
Adipocytokine signaling pathway	0.921196545283092	1.839969011
Signaling pathways regulating pluripotency of stem cells	0.674024638737121	1.830485931
Cell cycle	0.703390070731073	1.80853872
Endometrial cancer	1.00121285844965	1.807937943
GnRH signaling pathway	0.812639675637535	1.801243874
cGMP-PKG signaling pathway	0.597703725733847	1.796723547
Insulin secretion	0.818193294231804	1.762452258
Fc epsilon RI signaling pathway	0.904664314978368	1.736118913
Oocyte meiosis	0.687384748749352	1.679721199
Axon guidance	0.557177531893221	1.676126758
Cytokine-cytokine receptor interaction	0.441940384009462	1.67217606
Glucagon signaling pathway	0.724553772156384	1.659818821
Longevity regulating pathway	0.724553772156384	1.659818821
Human papillomavirus infection	0.397660121629863	1.65830753
Bacterial invasion of epithelial cells	0.838472211960026	1.645743047
Gastric acid secretion	0.838472211960026	1.645743047
ErbB signaling pathway	0.776811741128923	1.607153262
Intestinal immune network for IgA production	1.06412606536533	1.601962698
Hematopoietic cell lineage	0.727400885357647	1.579116893
Adrenergic signaling in cardiomyocytes	0.581713282037688	1.563050365
Tuberculosis	0.532567696581932	1.562684613
Gap junction	0.730833158190224	1.497603529
IL-17 signaling pathway	0.707573076899093	1.486468092
Melanoma	0.791104172114158	1.486078066
Prolactin signaling pathway	0.791104172114158	1.486078066
Gastric cancer	0.556226757861551	1.483269896
AMPK signaling pathway	0.593910102230119	1.447681928
Fc gamma R-mediated phagocytosis	0.708377890547675	1.445289381
Endocytosis	0.410650520799807	1.438404287
Salivary secretion	0.735029080692873	1.415142538

Term	log2FoldChange	-Log10(pvalue)
Arginine and proline metabolism	0.90790429916245	1.411890225
Central carbon metabolism in cancer	0.803651934963353	1.404411055
Acute myeloid leukemia	0.76834977050353	1.387419014
Fluid shear stress and atherosclerosis	0.535059348825809	1.366613985
Apelin signaling pathway	0.544031719352161	1.365676563
Ubiquitin mediated proteolysis	0.544031719352161	1.365676563
Cushing syndrome	0.499566217684824	1.336106198
Choline metabolism in cancer	0.627427726574016	1.334651892
Sphingolipid signaling pathway	0.559771873634829	1.325287742

Table S4. Genes associated to the highlighted KEGG pathways, related to Figure 4.

Th1 and Th2 cell differentiation	Th17 cell differentiation	JAK-STAT signaling pathway	T cell receptor signaling pathway
CD3D	AHR	AKT2	AKT2
FOS	CD3D	AKT3	AKT3
GATA3	FOS	BCL2L1	CARD11
H2-AA	GATA3	CCND2	CBLB
IL12A	H2-AA	CCND3	CD3D
IL13	HIF1A	CDKN1A	CD8B1
IL2	IL17D	CISH	CDC42
IL2RA	IL1RAP	CRLF2	FOS
IL2RB	IL2	CSF2RA	FYN
IL4	IL21R	EGFR	GRAP2
IL4RA	IL2RA	GRB2	GRB2
IL5	IL2RB	IFNAR1	IL10
JAK2	IL4	IFNAR2	IL2
JAK3	IL4RA	IFNK	IL4
JUN	IL6RA	IL10	IL5
LAT	IL6ST	IL10RB	JUN
LCK	IRF4	IL12A	KRAS
MAF	JAK2	IL13	LAT
MAML1	JAK3	IL15RA	LCK
MAML3	JUN	IL17D	LCP2
NFATC1	LAT	IL2	MALT1
NFATC2	LCK	IL20	MAP3K7
NFATC3	NFATC1	IL20RB	MAP3K8
NFKB1	NFATC2	IL21R	NCK2
NFKBIA	NFATC3	IL24	NFATC1
NOTCH1	NFKB1	IL2RA	NFATC2
NOTCH2	NFKBIA	IL2RB	NFATC3
PLCG1	PLCG1	IL3RA	NFKB1
PPP3CC	PPP3CC	IL4	NFKBIA
PRKCQ	PRKCQ	IL4RA	PIK3CD
RBPJ	RORA	IL5	PLCG1
RBPJL	RUNX1	IL6RA	PPP3CC
RUNX3	SMAD3	IL6ST	PRKCQ
STAT1	STAT1	IL7	PTPRC
STAT4	STAT3	IL7R	RAF1
STAT5B	STAT5B	JAK2	RASGRP1
TBX21	TBX21	JAK3	TEC
TYK2	TGFB1	LIF	TNF
ZAP70	TGFBR1	MCL1	VAV1
	TGFBR2	MYC	ZAP70
	TYK2	OSMR	
	ZAP70	PDGFB	
		PIAS2	
		PIAS4	
		PIK3CD	
		PIM1	
		PRLR	
		PTPN2	
		RAF1	
		SOCS1	
		SOCS6	
		STAM2	
		STAT1	
		STAT2	
		STAT3	
		STAT4	
		STAT5B	
		TYK2	

Table S6. List of genes associated with ADNP-CHD4-BRG1 peaks and with reduced H3K27ac in ADNP-deficient Th2 cells, related to Figure 7.

Il13	*Pstpip1*	*Mxi1*	*Cysltr2*
Il4ra	*Cyp4f39*	*Dusp6*	*Saysd1*
Maf	*Inpp5a*	*Rab27a*	*Bcl2l14*
Pdgfb	*Psap*	*Nfyb*	*Stim1*
Tmem40	*Usp37*	*Fosl2*	*Rbpj*
Pth	*Tmsb10*	*Ak2*	*Ankrd50*
Gm42602	*Stat5b*	*Cpd*	*Gm47824*
Slc44a2	*Fgf7*	*Ammecr1l*	*Dcp1b*
B3galt2	*Dusp11*	*Tsen2*	*Gm37176*
Gse1	*Tacc1*	*Ccdc71l*	*Alpk2*
Birc3	*Acot11*	*Jak2*	*S1pr1*
Egln3	*Gm35808*	*Atg10*	*Rtkn2*
Id2	*Gm49337*	*Mbnl1*	*Srgap3*
Srf	*Rbm47*	*Cyp11a1*	*Cbr4*
Igfbp4	*Ptpn1*	*Gm43822*	*Cd83*
Adora2b	*Sft2d2*	*Znrf2*	*Gm9887*
Vmp1	*Ern1*	*Arhgap12*	*Rapgef5*
Slco3a1	*Heg1*	*Gm44068*	*Mrpl39*
Rnf19b	*B4galt5*	*Samhd1*	*Pde4b*
Inhba	*Mapk6*	*Efna5*	*4930412O13Rik*
Nxpe3	*Irf2bp2*	*Kcnk5*	*Zfp36l2*
Ccl17	*Pparg*	*Ccr4*	*4931412I15Rik*
Sesn2	*Ddit4*	*Acsl4*	*Gm44627*
Dysf	*Hsf2bp*	*Ddr1*	*Tex13c2*
Map2k6	*Nfil3*	*Ttc30a1*	*Dnajc1*
Ninj1	*Gpx3*	*E130114P18Rik*	*Cpm*
Slc35a5	*Rnf19a*	*Gm44861*	*Gtdc1*
Manbal	*Muc13*	*Ndfip1*	*Ebf1*
Nab2	*Gm43963*	*Ankrd46*	*Gm47089*
Gm37352	*Ripor2*	*Gm38341*	*Gc*
Dcakd	*Celsr1*	*Rnf152*	*Gm17359*
Arf2	*Ywhah*	*Lpp*	*Thada*
Il24	*Zbtb38*	*Gcm1*	*AL607142.1*
Gm42906	*F2rl1*	*Rpl11*	*Kcnj15*
Rps6ka5	*Gm44062*	*Nsmce2*	*Ube2cbp*
Cd200	*Tns4*	*Col18a1*	*Cstf2t*
Kif3b	*Stk33*	*Gramd1b*	*Cd47*
Gm37535	*Vgll4*	*Rassf6*	*Samsn1*
Wdfy2	*Gcg*	*Kbtbd11*	*Gm49167*
Map3k3	*Lactb*	*Laptm4a*	*Rasgrp1*
Itga3	*Nckap5*	*Utp23*	*Tomm20*
Batf	*Adamtsl4*	*Ube2v1*	*Zfpm1*
Rab13	*Ier5*	*Med13l*	*Atg7*
Mdm2	*Sec61g*	*Bcat1*	*Jun*
Cmss1	*Gm44198*	*Gm43636*	*Chd7*
Anp32a	*Asb2*	*Notch2*	*Nup35*
Erc1	*Ksr1*	*Gm43637*	*Gja1*
Elk3	*Ncoa3*	*Pde11a*	*Jmjd1c*
Kat7	*Smndc1*	*Vstm5*	*3110018I06Rik*
Rcc1	*Jade2*	*Fam49b*	*Myc*
Uqcrfs1	*Samd13*	*Septin9*	*Mllt3*
Pim3	*Cnst*	*Gm37192*	*Runx1*
Pecam1	*Ier3*	*Auh*	*Smyd3*
Tmem154	*Stoml1*	*Eif4e*	*Rnf144a*
Sox5	*Rnf157*	*Dnajc5b*	*Rab3c*
Thy1	*Klhl25*	*Coro6*	*Gsdmc*
Sema4d	*Jarid2*	*Gm13691*	*Gm48502*
